# Subtraction-Average-Based Optimizer: A New Swarm-Inspired Metaheuristic Algorithm for Solving Optimization Problems

**DOI:** 10.3390/biomimetics8020149

**Published:** 2023-04-06

**Authors:** Pavel Trojovský, Mohammad Dehghani

**Affiliations:** Department of Mathematics, Faculty of Science, University of Hradec Králové, 500 03 Hradec Králové, Czech Republic; mohammad.dehghani@uhk.cz

**Keywords:** optimization, swarm-inspired, metaheuristic, subtraction average, exploration, exploitation

## Abstract

This paper presents a new evolutionary-based approach called a Subtraction-Average-Based Optimizer (SABO) for solving optimization problems. The fundamental inspiration of the proposed SABO is to use the subtraction average of searcher agents to update the position of population members in the search space. The different steps of the SABO’s implementation are described and then mathematically modeled for optimization tasks. The performance of the proposed SABO approach is tested for the optimization of fifty-two standard benchmark functions, consisting of unimodal, high-dimensional multimodal, and fixed-dimensional multimodal types, and the CEC 2017 test suite. The optimization results show that the proposed SABO approach effectively solves the optimization problems by balancing the exploration and exploitation in the search process of the problem-solving space. The results of the SABO are compared with the performance of twelve well-known metaheuristic algorithms. The analysis of the simulation results shows that the proposed SABO approach provides superior results for most of the benchmark functions. Furthermore, it provides a much more competitive and outstanding performance than its competitor algorithms. Additionally, the proposed approach is implemented for four engineering design problems to evaluate the SABO in handling optimization tasks for real-world applications. The optimization results show that the proposed SABO approach can solve for real-world applications and provides more optimal designs than its competitor algorithms.

## 1. Introduction

Optimization is a comprehensive concept in various fields of science. An optimization problem is a type of problem that has more than one feasible solution. Therefore, the goal of optimization is to find the best solution among all these feasible solutions. From a mathematical point of view, an optimization problem is explained using three parts: decision variables, constraints, and objective function [1]. The problem solving techniques in optimization studies are placed into two groups: deterministic and stochastic approaches [2].

Deterministic approaches, which are placed into two classes, gradient-based and non-gradient-based, are effective in solving linear, convex, simple, low-dimensional, continuous, and differentiable optimization problems [3]. However, increasing the complexity of these optimization problems leads to disruption in the performance of the deterministic approaches, and these methods get stuck in inappropriate local optima. On the other hand, many optimization problems within science and real-world applications have characteristics such as a high dimensionality, a high complexity, a non-convex, non-continuous, non-linear, and non-differentiable objective function, and a non-linear and unknown search space [4]. These optimization task characteristics and the difficulties of deterministic approaches have led researchers to introduce new techniques called stochastic approaches.

Metaheuristic algorithms are one of the most widely used stochastic approaches that effectively solve complex optimization problems. They have efficiency in solving non-linear, non-convex, non-differentiable, high-dimensional, and NP-hard optimization problems. An efficiency in addressing discrete, non-linear, and unknown search spaces, the simplicity of their concepts, their easy implementation, and their non-dependence on the type of problem are among the advantages that have led to the popularity of metaheuristic algorithms [5]. Metaheuristic algorithms are employed in various optimization applications within science, such as index tracking [6], energy [7,8,9,10], protection [11], energy carriers [12,13], and electrical engineering [14,15,16,17,18,19].

The optimization process of these metaheuristic algorithms is based on random search in the problem solving space and the use of random operators. Initially, candidate solutions are randomly generated. Then, during a repetition-based process and based on the steps of the algorithm, to improve the quality of these initial solutions, the position of the candidate solutions in the problem solving space is updated. In the end, the best candidate solution is available to solve the problem. Using random search in the optimization process does not guarantee the achievement of the global optimal by a metaheuristic algorithm. For this reason, the solutions that are obtained from metaheuristic algorithms are called pseudo-optimal [20]. To organize an effective search in the problem solving space, metaheuristic algorithms should be able to provide and manage search operations well, at both global and local levels. Global search, with the concept of exploration, leads to a comprehensive search in the problem solving space and an escape from optimal local areas. Local search, with the concept of exploitation, leads to a detailed search around the promising solutions for a convergence towards possible better solutions. Considering that exploration and exploitation pursue opposite goals, the key to the success of metaheuristic algorithms is to create a balance between this exploration and exploitation during the search process [21].

On the one hand, the concepts of the random search process and quasi-optimal solutions, and, on the other hand, the desire to achieve better quasi-optimal solutions for these optimization problems, have led to the development of numerous metaheuristic algorithms by researchers.

The main research question is that now that many metaheuristic algorithms have been designed, is there still a need to introduce a newer algorithm to deal with optimization problems or not? In response to this question, the No Free Lunch (NFL) [22] theorem explains that the high success of a particular algorithm in solving a set of optimization problems will not guarantee the same performance of that algorithm for other optimization problems. There is no assumption that implementing an algorithm on an optimization problem will be successful. According to the NFL theorem, no particular metaheuristic algorithm is the best optimizer for solving all optimization problems. The NFL theorem motivates researchers to search for better solutions for these optimization problems by designing newer metaheuristic algorithms. The NFL theorem has also inspired the authors of this paper to provide more effective solutions for dealing with optimization problems by creating a new metaheuristic algorithm.

The innovation and novelty of this paper are in the introduction a new metaheuristic algorithm called the Subtraction Average of Searcher Agents (SABO) for solving the optimization problems in different sciences. The main contributions of this study are as follows:

- The basic idea behind the design of the SABO is the mathematical concepts and information subtraction average of the algorithm’s search agents.
- The steps of the SABO’s implementation are described and its mathematical model is presented.
- The efficiency of the proposed SABO approach has been evaluated for fifty-two standard benchmark functions.
- The quality of the SABO’s results has been compared with the performance of twelve well-known algorithms.
- To evaluate the capability of the SABO in handling real-world applications, the proposed approach is implemented for four engineering design problems.

The continuation of this paper is organized as follows: the literature review is presented in Section 2. The proposed SABO approach is introduced and designed in Section 3. Its simulation studies are presented in Section 4. The performance of the SABO in solving real-world applications is evaluated in Section 5. The conclusions and several research suggestions are provided in Section 6.

## 2. Literature Review

Metaheuristic algorithms have been developed with inspiration from various natural phenomena, the behaviors of living organisms in nature, concepts of biology, physical sciences, rules of games, and human interactions, etc. In a general classification based on the idea that is employed in their design, metaheuristic algorithms are placed into five groups: swarm-based, evolutionary-based, physics-based, human-based, and game-based approaches.

Swarm-based metaheuristic algorithms are approaches that are inspired by various natural swarming phenomena, such as the natural behaviors of animals, birds, aquatic animals, insects, and other living organisms. Among the most famous swarm-based approaches are particle swarm optimization (PSO) [23], ant colony optimization (ACO) [24], and artificial bee colony (ABC) [25]. PSO is a swarming method that is inspired by the movement strategy of flocks of fish or birds searching for food in nature. ACO is inspired by ant colonies’ ability to choose the shortest path between the food source and the colony site. ABC is derived from the hierarchical strategy of honey bee colonies and their activities in finding food sources. The strategies of providing food through hunting and foraging, migration, and the process of chasing between living organisms are some of the most natural, characteristic swarming ways of behavior, which have been a source of inspiration in the design of numerous swarm-based algorithms, such as the Reptile Search Algorithm (RSA) [26], Orca Predation Algorithm (OPA) [27], Marine Predator Algorithm (MPA) [28], African Vultures Optimization Algorithm (AVOA) [29], Honey Badger Algorithm (HBA) [30], White Shark Optimizer (WSO) [31], Whale Optimization Algorithm (WOA) [32], Tunicate Swarm Algorithm (TSA) [33], Grey Wolf Optimizer (GWO) [34], and Golden Jackal Optimization (GJO) [35].

Evolutionary-based metaheuristic algorithms are approaches that are developed based on simulating the concepts of the biological and genetic sciences. The bases of these methods are evolution strategies (ES) [36], genetic algorithms (GA) [37], and differential evolution (DE) [36]. These methods and all their generalizations are inspired by the concepts of biology, natural selection, Darwin’s theory of evolution, reproduction, and stochastic operators such as selection, crossover, and mutation.

Physics-based metaheuristic algorithms are designed based on modeling phenomena, processes, concepts, and the different forces in physics. Simulated annealing (SA) [38] is one of the most widely used physics-based methods, whose design is inspired by the annealing process of metals. In the annealing process, the metal is first melted under heat, then gradually cooled to achieve the ideal crystal. The modeling of physical forces and the laws of motion is the design origin of physics-based algorithms such as the gravitational search algorithm (GSA) [39] and momentum search algorithm (MSA) [40]. SA is developed based on the modeling of the tensile force and Hooke’s law between bodies that are connected by springs. Gravitational force inspires the GSA, which masses at the different distances that exert on each other. The MSA is designed based on the modeling of the force that results from the momentum of balls that hit each other. The phenomenon of the transformations of different physical states in the natural water cycle is employed in the water cycle algorithm’s (WCA) [41] design. The concepts of cosmology and black holes have been the primary sources for the design of algorithms such as the Black Hole Algorithm (BHA) [42] and Multi-Verse Optimizer (MVO) [43]. Some of the other physics-based algorithms are: the Equilibrium Optimizer (EO) [44], Thermal Exchange Optimization (TEO) [45], the Archimedes optimization algorithm (AOA) [46], the Lichtenberg Algorithm (LA) [47], Henry Gas Optimization (HGO) [48], Electro-Magnetism Optimization (EMO) [49], and nuclear reaction optimization (NRO) [50].

Human-based metaheuristic algorithms are approaches with designs that are inspired by the interactions, relationships, and thoughts of humans in social and individual life. Teaching–learning-based optimization (TLBO) [51] is one of the most familiar and widely used human-based approaches, whose design is inspired by the scientific interactions between teachers and students in the educational system. The effort of two social classes, the poor and rich, to improve their economic situations was the main idea behind introducing poor and rich optimization (PRO) [45]. The cooperation and interactions between teammates within a team to achieve their set goal has been the main idea behind the introduction of the Teamwork Optimization Algorithm (TOA) [52]. Collective decision optimization (CDO) [45] is inspired by the decision making behavior of humans, the queuing search algorithm (QSA) [45] mimics human actions when performing a queuing process, the political optimizer (PO) [50] imitates a human political formwork, and the Election-Based Optimization Algorithm (EBOA) [45] is based on mimicking the voting process for leader selections. Some of the other human-based algorithms are, e.g., the gaining–sharing knowledge-based algorithm (GSK) [53], Ali Baba and the Forty Thieves (AFT) [54], Driving Training-Based Optimization (DTBO) [4], the Coronavirus herd immunity optimizer (CHIO) [55], and War Strategy Optimization (WSO) [56].

Game-based metaheuristic algorithms are approaches that are introduced based on modeling the rules of different individual and group games and the strategies of their players, coaches, referees, and the other people influencing the games. Football and volleyball are popular group games whose simulations have been employed in the design of the League Championship Algorithm (LCA) [57], Volleyball Premier League (VPL) [57], and Football-Game-Based Optimization (FGBO) [58], respectively.

Mathematics-based metaheuristic algorithms are designed based on mathematical concepts, foundations, and operations. The Sine Cosine Algorithm (SCA) [51] is one of the most familiar mathematics-based approaches, whose design is inspired by the transcendental functions sin and cos. The Arithmetic Optimization Algorithm (AOA) [51] uses the distribution behavior of mathematics’ four basic arithmetic operators (multiplication, division, subtraction, and addition). Runge Kutta (RUN) [51] uses the logic of slope variations that are computed by the RK method as a promising and logical searching mechanism for global optimization. The Average and Subtraction-Based Optimizer (ASBO) [51] has the main construction idea of computing the averages and subtractions of the best and worst population members for guiding the algorithm population in the problem search space.

Based on the best knowledge from the literature review, no metaheuristic algorithm has been developed based on the mathematical concept of “an average of subtraction of search agents”. Therefore, the primary idea of the proposed algorithm was to use an extraordinary average of all the search agents to update the algorithm’s population, which can prevent the algorithm’s dependence on specific population members. Moreover, by improving the exploration of the algorithm, this can avoid it getting stuck in local optima. Therefore, to address this research gap in optimization studies, in this paper, a new metaheuristic algorithm is designed based on the mathematical concept of a special subtraction arithmetic average, which is discussed in the next section.

## 3. Subtraction-Average-Based Optimizer

In this section, the theory of the proposed Subtraction-Average-Based Optimizer (SABO) approach is explained, then its mathematical modeling is presented for its employment in optimization tasks.

### 3.1. Algorithm Initialization

Each optimization problem has a solution space, which is called the search space. The search space is a subset of the space of the dimension, which is equal to the number of the decision variables of the given problem. According to their position in the search space, algorithm searcher agents (i.e., population members) determine the values for the decision variables. Therefore, each search agent contains the information of the decision variables and is mathematically modeled using a vector. The set of search agents together forms the population of the algorithm. From a mathematical point of view, the population of the algorithm can be represented using a matrix, according to Equation (1). The primary positions of the search agents in the search space are randomly initialized using Equation (2).

(1) $$X={\left[\begin{array}{c} X_{1}\\ \vdots \\ X_{i}\\ \vdots \\ X_{N} \end{array}\right]}_{N\times m}={\left[\begin{array}{ccccc} x_{1,1} & \cdots & x_{1,d} & \cdots & x_{1,m}\\ \vdots & \ddots & \vdots & ⋰ & \vdots \\ x_{i,1} & \cdots & x_{i,d} & \cdots & x_{i,m}\\ \vdots & ⋰ & \vdots & \ddots & \vdots \\ x_{N,1} & \cdots & x_{N,d} & \cdots & x_{N,m} \end{array}\right]}_{N\times m},$$

(2) $$x_{i,d}=lb_{d}+r_{i,d}\cdot \left(ub_{d}-lb_{d}\right),\;i=1,\ldots ,N,\;d=1,\ldots ,m,$$

where X is the SABO population matrix, *X_i_* is the ith search agent (population member), $x_{i,d}$ is its dth dimension in the search space (decision variable), N is the number of search agents, m is the number of decision variables, $r_{i,d}$ is a random number in the interval [0,1], and $lb_{d}$ and $ub_{d}$ are the lower and upper bounds of the dth decision variable, respectively.

Each search agent is a candidate solution to the problem that suggests values for the decision variables. Therefore, the objective function of the problem can be evaluated based on each search agent. The evaluated values for the objective function of the problem can be represented by using a vector called $\vec{F}$, according to Equation (3). Based on the placement of the specified values by each population member for the decision variables of the problem, the objective function is evaluated and stored in the vector $\vec{F}$. Therefore, the number of elements of the vector $\vec{F}$ is equal to the number of the population members N.

(3) $$\vec{F}={\left[\begin{array}{c} F_{1}\\ \vdots \\ F_{i}\\ \vdots \\ F_{N} \end{array}\right]}_{N\times 1}={\left[\begin{array}{c} F\left(X_{1}\right)\\ \vdots \\ F\left(X_{i}\right)\\ \vdots \\ F\left(X_{N}\right) \end{array}\right]}_{N\times 1},$$

where $\vec{F}$ is the vector of the values of the objective function, and $F_{i}$ is the evaluated values for the objective function based on the ith search agent.

The evaluated values for the objective function are a suitable criterion for analyzing the quality of the solutions that are proposed by the search agents. Therefore, the best value that is calculated for the objective function corresponds to the best search agent. Similarly, the worst value that is calculated for the objective function corresponds to the worst search agent. Considering that the position of the search agents in the search space is updated in each iteration, the process of identifying and saving the best search agent continues until the last iteration of the algorithm.

### 3.2. Mathematical Modelling of SABO

The basic inspiration for the design of the SABO is mathematical concepts such as averages, the differences in the positions of the search agents, and the sign of difference of the two values of the objective function. The idea of using the arithmetic mean location of all the search agents (i.e., the population members of the tth iteration), instead of just using, e.g., the location of the best or worst search agent to update the position of all the search agents (i.e., the construction of all the population members of the (t+1)th iteration), is not new, but the SABO’s concept of the computation of the arithmetic mean is wholly unique, as it is based on a special operation “${-}_{v}$”, called the v−subtraction of the search agents B from the search agent A, which is defined as follows:

(4) $$A\;{-}_{v}\;B=\mathrm{sign}\;\left(F\left(A\right)-F\left(B\right)\right)\left(A-\vec{v}*B\right),$$

where $\vec{v}$ is a vector of the dimension m, in which components are random numbers that are generated from the set {1,2}, the operation “∗” represents the Hadamard product of the two vectors (i.e., all the components of the resulting vectors are formed by multiplying the corresponding components of the given two vectors), F(A) and F(B) are the values of the objective function of the search agents A and B, respectively, and sign is the signum function. It is worth noting that, due to the use of a random vector $\vec{v}$ with components from the set {1,2} in the definition of the v−subtraction, the result of this operation is any of the points of a subset of the search space that has a cardinality of $2^{m+1}$.

In the proposed SABO, the displacement of any search agent $X_{i}$ in the search space is calculated by the arithmetic mean of the v−subtraction of each search agent $X_{j},\;j=1,2,\ldots ,N,$ from the search agent $X_{i}$. Thus, the new position for each search agent is calculated using (5).

(5) $$X_{i}^{new}=X_{i}+{\vec{r}}_{i}*\frac{1}{N}\overset{N}{\underset{j=1}{\sum }}\left(X_{i}\;\;{-}_{v}\;X_{j}\right),\;\;i=1,2,\;\ldots ,\;N,$$

where $X_{i}^{new}$ is the new proposed position for the ith search agent $X_{i}$, N is the total number of the search agents, and ${\vec{r}}_{i}$ is a vector of the dimension m, in which components have a normal distribution with the values from the interval [0, 1].

Then, if this proposed new position leads to an improvement in the value of the objective function, it is acceptable as the new position of the corresponding agent, according to (6).

(6) $$X_{i}=\{\begin{array}{cc} X_{i}^{new}, & \;F_{i}^{new}<F_{i};\\ X_{i}, & \;else, \end{array}$$

where $F_{i}$ and $F_{i}^{new}$ are the objective function values of the search agents $X_{i}$ and $X_{i}^{new},$ respectively.

**Figure 1:**
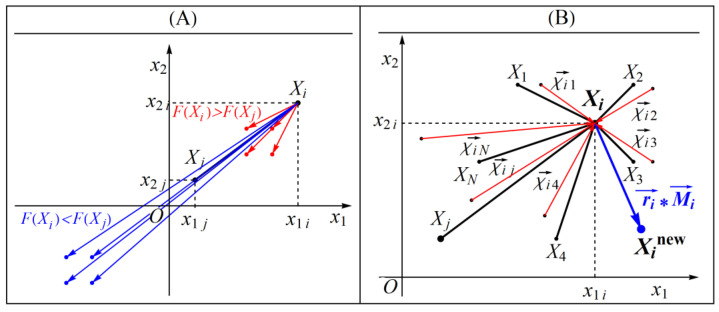
Schematic illustration (for the case m=2) of the exploration phase by “v -subtraction” (**A**), and the exploitation phase by “arithmetic mean of the v -subtractions” (**B**).

Clearly, the v−subtraction $X_{i}\;\;{-}_{v}\;X_{j}$ represents a vector ${\vec{\chi }}_{ij},$ and we can look at Equation (5) as the motion equation of the search agent $X_{i}$, since we can rewrite it in the form $X_{i}^{new}=X_{i}+{\vec{r}}_{i}*{\vec{M}}_{i}$, where the mean vector ${\vec{M}}_{i}=\frac{1}{N}\overset{N}{\underset{j=1}{\sum }}\left(X_{i}\;\;{-}_{v}\;X_{j}\right)=\frac{1}{N}\overset{N}{\underset{j=1}{\sum }}{\vec{\chi }}_{ij}$ determines the direction of the movement of the search agent $X_{i}$ to its new position $X_{i}^{new}.$ The search mechanism based on “the arithmetic mean of the v-subtractions”, which is presented in (5), has the essential property of realizing both the exploration and exploitation phases to explore the promising areas in the search space. The exploration phase is realized by the operation of “v-subtraction” (i.e., the vector ${\vec{\chi }}_{ij}$), see Figure 1A, and the exploitation phase by the operation of the “arithmetic mean of the v-subtractions” (i.e., the vector ${\vec{M}}_{i}$), see Figure 1B.

### 3.3. Repetition Process, Pseudocode, and Flowchart of SABO

**Figure 2:**
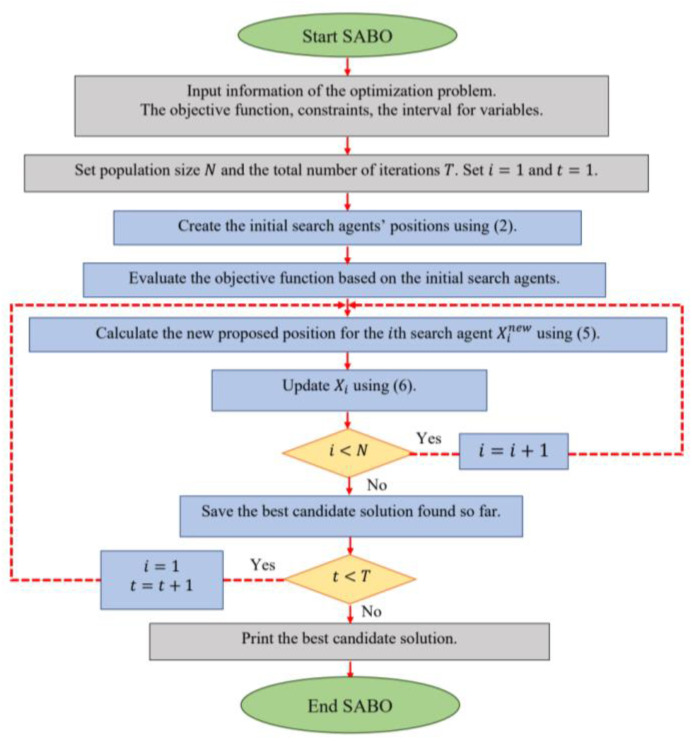
Flowchart of SABO.

After updating all the search agents, the first iteration of the algorithm is completed. Then, based on the new values that have been evaluated for the positions of the search agents and objective function, the algorithm enters its next iteration. In each iteration, the best search agent is stored as the best candidate solution so far. This process of updating the search agents continues until the last iteration of the algorithm, based on (3) to (5). In the end, the best candidate solution that was stored during the iterations of the algorithm is presented as the solution to the problem. The implementation steps of the SABO are shown as a flowchart in Figure 2 and presented as a pseudocode in Algorithm 1.
**Algorithm 1.** Pseudocode of SABO.Start SABO.1. Input problem information: variables, objective function, and constraints.2. Set SABO population size (*N*) and iterations (*T*).3. Generate the initial search agents’ matrix at random using Equation (2). $x_{i,d}\leftarrow lb_{d}+r_{i,d}\cdot \left(ub_{d}-lb_{d}\right)$4. Evaluate the objective function.5.   For *t* = 1 to *T*6.     For *i* = 1 to N7.    Calculate new proposed position for *i*th SABO search agent using Equation (5). $x_{i,d}^{new}\leftarrow X_{i}+{\vec{r}}_{i}*\frac{1}{N}\overset{N}{\underset{j=1}{\sum }}\left(X_{i}\;\;{-}_{v}\;X_{j}\right)$8.    Update *i*th GAO member using Equation (6). $X_{i}\leftarrow \{\begin{array}{cc} X_{i}^{new}, & \;\;F_{i}^{new}<F_{i}\\ X_{i}, & \;\;else \end{array}$9.     end10.   Save the best candidate solution so far.11.   end12. Output best quasi-optimal solution obtained with the SABO.End SABO.

### 3.4. Computational Complexity of SABO

In this subsection, the computational complexity of the proposed SABO approach is evaluated. The initialization steps of the SABO for dealing with an optimization problem with m decision variables have a complexity that is equal to O(Nm), where N is the number of search agents. Furthermore, the process of updating these search agents has a complexity that is equal to O(NmT), where T is the total number of iterations of the algorithm. Therefore, the computational complexity of the SABO is equal to O(Nm(1+T)).

## 4. Simulation Studies and Results

In this section, the effectiveness of the proposed SABO approach in solving optimization problems is challenged. For this purpose, a set of fifty-two standard benchmark functions is employed, consisting of seven unimodal functions (F1 to F7), six high-dimensional multimodal functions (F8 to F13), ten fixed-dimensional multimodal functions (F14 to F23), and twenty-nine functions from the CEC 2017 test suite [59]. To analyze the performance quality of the SABO in optimization tasks, the results that were obtained from the proposed approach have been compared with twelve well-known metaheuristic algorithms: GA, PSO, GSA, TLBO, GWO, MVO, WOA, MPA, TSA, RSA, WSO, and AVOA. The values of the control parameters of the competitor algorithms are specified in Table 1.

The proposed SABO and each of the competitor algorithms are implemented for twenty independent runs on the benchmark functions, where each independent run includes 1000 iterations. The optimization results are reported using six indicators: the mean, best, worst, standard deviation (std), median, and rank. The ranking criterion of these metaheuristic algorithms is based on providing a better value for the mean index.

### 4.1. Evaluation Unimodal Functions

The unimodal objective functions, F1 to F7, due to the lack of local optima, are suitable options for analyzing the exploitation ability of the metaheuristic algorithms. The optimization results of the F1 to F7 functions, using the SABO and competitor algorithms, are reported in Table 2.

Based on the obtained results, the proposed SABO, with a high exploitation ability, provided the global optimal when solving the F1, F2, F3, F4, and F6 functions. Additionally, the SABO is the best optimizer for the F5 and F7 functions. A comparison of the simulation results shows that the SABO, through obtaining the first rank in the total, provided a superior performance for solving the unimodal problems F1 to F7 compared to the competitor algorithms.

### 4.2. Evaluation High-Dimensional Multimodal Functions

The high-dimensional multimodal objective functions, F8 to F13, due to having a large number of local optima, are suitable options for evaluating the exploration ability of the metaheuristic algorithms. The results of implementing the SABO and its competitor algorithms on the functions F8 to F13 are reported in Table 3.

Based on the optimization results, the SABO provided the global optimal for the F9 and F11 functions, with a high exploration ability. The proposed SABO approach is the best optimizer for solving the functions F8, F10, F12, and F13. The analysis of the simulation results shows that the SABO provided a superior performance in handling the high-dimensional multimodal problems compared to its competitor algorithms.

### 4.3. Evaluation Fixed-Dimensional Multimodal Functions

The fixed-dimensional multimodal objective functions, F14 to F23, have fewer numbers of local optima than the functions F8 to F13. These functions are suitable options for evaluating the ability of the metaheuristic algorithms to create a balance between the exploration and exploitation. The optimization results of the operations F14 to F23, using the SABO and its competitor algorithms, are presented in Table 4.

Based on the obtained results, the SABO is the best optimizer for the functions F15 and F21. In solving the other benchmark functions of this group, the SABO had a similar situation to some of its competitor algorithms from the point of view of the mean criterion. However, the proposed SABO algorithm performed better in solving these functions by providing better values for the std index. Furthermore, the analysis of the simulation results shows that, compared to the competitor algorithms, the SABO provided a superior performance by balancing the exploration and exploitation in the optimization of the fixed-dimensional multimodal problems.

**Figure 3:**
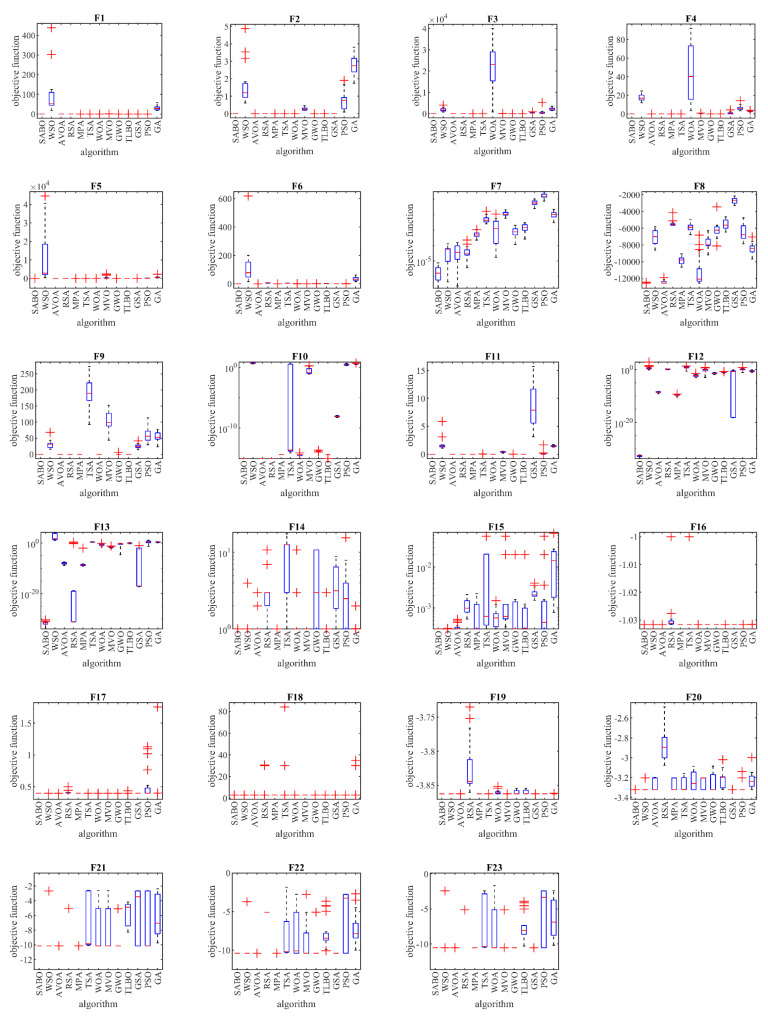
Boxplot diagrams of the proposed SABO and competitor algorithms for F1 to F23 test functions.

The performances of the proposed SABO approach and the competitor algorithms in solving the functions F1 to F23 are presented in the form of boxplot diagrams in Figure 3.

### 4.4. Evaluation CEC 2017 Test Suite

In this subsection, the efficiency of the SABO in solving the complex optimization problems from the CEC 2017 test suite is evaluated. The CEC 2017 test suite has thirty benchmark functions, consisting of three unimodal functions, C17-F1 to C17-F3, seven multimodal functions, C17-F4 to C17-F10, ten hybrid functions, C17-F11 to C17-F20, and ten composition functions, C17-F21 to C17-F30. The C17-F2 function was removed from this test suite due to its unstable behavior. The complete information on the CEC 2017 test suite is provided by [59]. The results of implementing the proposed SABO approach and its competitor algorithms on the CEC 2017 test suite are reported in Table 5.

**Figure 4:**
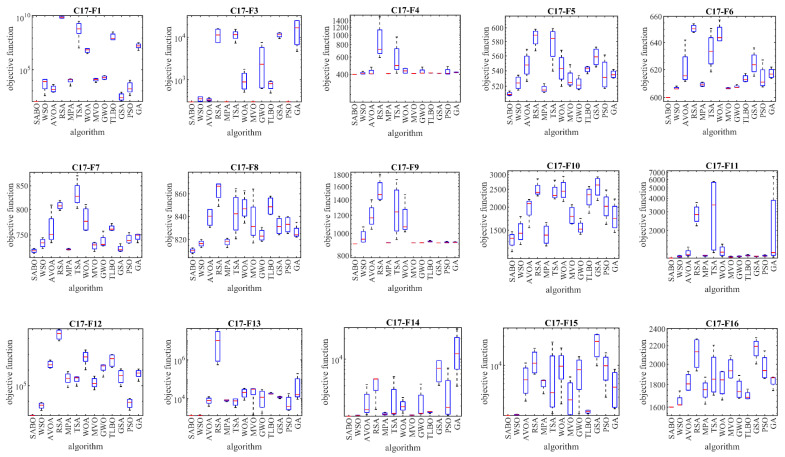

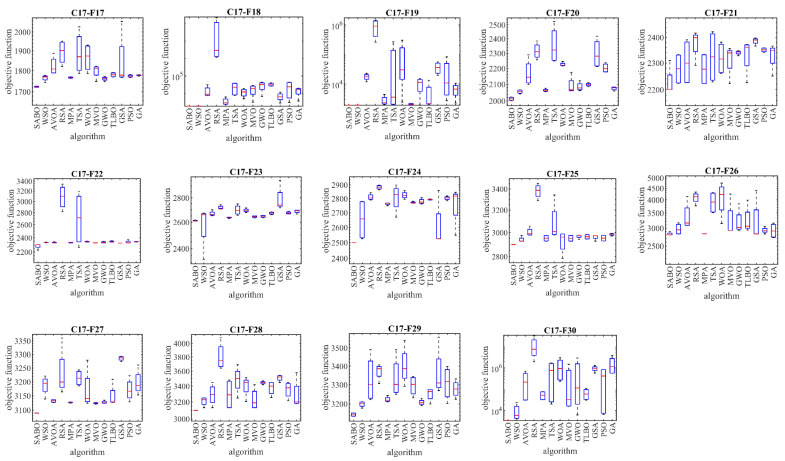
Boxplot diagram of SABO and competitor algorithms on the CEC 2017 test suite.

Based on the obtained results, the SABO is the best optimizer for the functions C17-F1, C17-F3 to C17-F23, and C17-F25 to C17-F30. The analysis of the simulation results shows that the proposed SABO approach provided better results for most of the benchmark functions. Overall, by winning the first rank, it provided a superior performance in handling the CEC 2017 test suite compared to the competitor algorithms. The performances of the SABO and its competitor algorithms in solving the CEC 2017 test suite are plotted as boxplot diagrams in Figure 4.

### 4.5. Statistical Analysis

In this subsection, statistical analyses are presented for the results of the proposed SABO approach and its competing algorithms to determine whether the superiority of the SABO over the competing algorithms is significant from a statistical point of view. For this purpose, the Wilcoxon rank sum test [60] was used, which is a non-parametric statistical analysis that is used to determine the significant difference between the averages of two data samples. In this test, an index called the p-value is used to determine the significant difference. The results of implementing the Wilcoxon rank sum test on the performances of the SABO and the competitor algorithms are presented in Table 6.

Based on the simulation results, in cases where the p-value was less than 0.05, the proposed SABO approach had a significant statistical superiority over the corresponding metaheuristic algorithm.

### 4.6. Advantages and Disadvantages of SABO

The proposed SABO approach is a metaheuristic algorithm that performs the optimization process based on the search power of its population through an iteration-based process. Among the advantages of the SABO, it can be mentioned that, except for the parameters of the number of population members N and the maximum number of iterations of the algorithm T, which are similar in all the algorithms, it does not have any control parameters. For this reason, it does not need a parameter-setting process. The simplicity of its equations, its easy implementation, and its simple concepts are other advantages of the SABO. The SABO’s ability to balance exploration and exploitation during the search process in the problem solving space is another advantage of this proposed approach. Despite these advantages, the proposed approach also has several disadvantages. The proposed SABO approach belongs to the group of stochastic techniques for solving optimization problems, and for this reason, its first disadvantage is that there is no guarantee of it achieving the global optimal. Another disadvantage of the SABO is that, based on the NFL theorem, it cannot be claimed that the proposed approach performs best in all optimization applications. Another disadvantage of the SABO is that there is always the possibility that newer metaheuristic algorithms will be designed that have a better performance than the proposed approach in handling some optimization tasks.

## 5. SABO for Real-World Applications

In this subsection, the capability of the proposed SABO approach in handling optimization tasks in real-world applications is challenged by four engineering design optimization problems.

### 5.1. Pressure Vessel Design Problem

**Figure 5:**
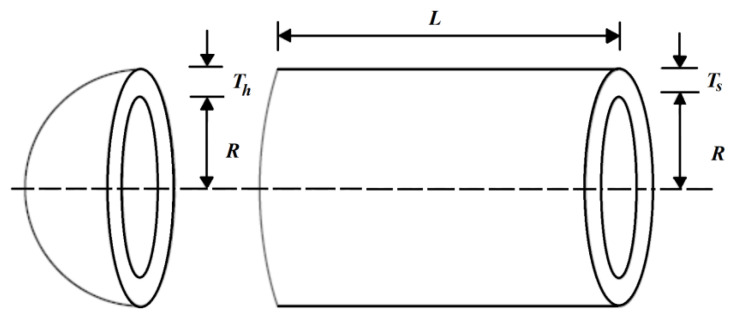
Schematic of the pressure vessel design.

The pressure vessel design is an optimization challenge with the aim of minimizing construction costs. The pressure vessel design schematic is shown in Figure 5.

The mathematical model of the pressure vessel design problem is as follows [61]:

Consider: $X=\left[x_{1},\;x_{2},\;x_{3},\;x_{4}\right]=\left[T_{s},\;T_{h},\;R,\;L\right]$.

Minimize: $f\left(x\right)=0.6224x_{1}x_{3}x_{4}+1.778x_{2}x_{3}^{2}+3.1661x_{1}^{2}x_{4}+19.84x_{1}^{2}x_{3}.$

Subject to:

$$g_{1}\left(x\right)=-x_{1}+0.0193x_{3}\;\leq \;0,\;g_{2}\left(x\right)=-x_{2}+0.00954x_{3}\leq \;0,\;$$

$$g_{3}\left(x\right)=-\pi x_{3}^{2}x_{4}-\frac{4}{3}\pi x_{3}^{3}+1296000\leq \;0,\;g_{4}\left(x\right)=x_{4}-240\;\leq \;0.$$

with

$$0\leq x_{1},x_{2}\leq 100\;\mathrm{and}\;10\leq x_{3},x_{4}\leq 200.$$

The optimization results for the pressure vessel design, using the SABO and its competing algorithms, are reported in Table 7 and Table 8.

**Figure 6:**
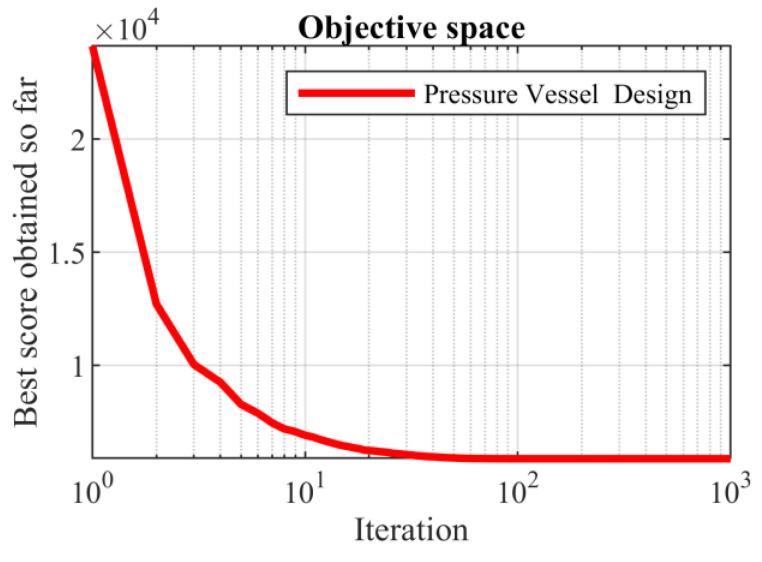
SABO’s performance convergence curve for the pressure vessel design.

Based on the obtained results, the SABO provided the optimal solution, with the values of the design variables being equal to (0.778027075, 0.384579186, 40.3122837, and 200) and the value of the objective function being equal to 5882.901334. The analysis of the simulation results shows that the SABO more effectively dealt with the pressure vessel design compared to its competing algorithms. The convergence curve of the SABO during the pressure vessel design optimization is drawn in Figure 6.

### 5.2. Speed Reducer Design Problem

**Figure 7:**
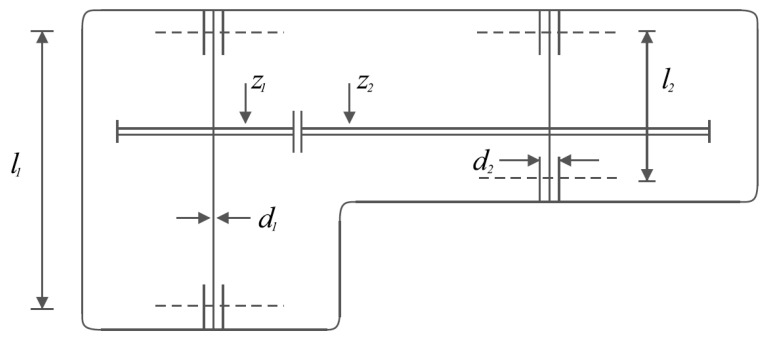
Schematic of the speed reducer design.

The speed reducer design is a real-world application within engineering science with the aim of minimizing the weight of the speed reducer. The speed reducer design schematic is shown in Figure 7.

The mathematical model of the speed reducer design problem is as follows [62,63]:

Consider: $X=\left[x_{1,}\;x_{2},\;x_{3},\;x_{4},\;x_{5},x_{6},x_{7}\right]=\left[b,\;m,\;p,\;l_{1},\;l_{2},\;d_{1},\;d_{2}\right]$.

Minimize: $f\left(x\right)=0.7854x_{1}x_{2}^{2}\left(3.3333x_{3}^{2}+14.9334x_{3}-43.0934\right)-1.508x_{1}\left(x_{6}^{2}+x_{7}^{2}\right)+7.4777\left(x_{6}^{3}+x_{7}^{3}\right)+0.7854\left(x_{4}x_{6}^{2}+x_{5}x_{7}^{2}\right)$.

Subject to:

$$g_{1}\left(x\right)=\frac{27}{x_{1}x_{2}^{2}x_{3}}-1\;\leq \;0,\;\;g_{2}\left(x\right)=\frac{397.5}{x_{1}x_{2}^{2}x_{3}}-1\leq \;0,$$

$$g_{3}\left(x\right)=\frac{1.93x_{4}^{3}}{x_{2}x_{3}x_{6}^{4}}-1\leq \;0,\;\;g_{4}\left(x\right)=\frac{1.93x_{5}^{3}}{x_{2}x_{3}x_{7}^{4}}-1\;\leq \;0,$$

$$g_{5}\left(x\right)=\frac{1}{110x_{6}^{3}}\sqrt{{\left(\frac{745x_{4}}{x_{2}x_{3}}\right)}^{2}+16.9\cdot 10^{6}}-1\leq \;0,$$

$$g_{6}\left(x\right)=\frac{1}{85x_{7}^{3}}\sqrt{{\left(\frac{745x_{5}}{x_{2}x_{3}}\right)}^{2}+157.5\cdot 10^{6}}-1\;\leq \;0,$$

$$g_{7}\left(x\right)=\frac{x_{2}x_{3}}{40}-1\;\leq \;0,\;\;g_{8}\left(x\right)=\frac{5x_{2}}{x_{1}}-1\;\leq \;0,$$

$$g_{9}\left(x\right)=\frac{x_{1}}{12x_{2}}-1\;\leq \;0,\;\;g_{10}\left(x\right)=\frac{1.5x_{6}+1.9}{x_{4}}-1\;\leq \;0.$$

$$g_{11}\left(x\right)=\frac{1.1x_{7}+1.9}{x_{5}}-1\;\leq \;0.$$

with

$$2.6\leq x_{1}\leq 3.6,\;0.7\leq x_{2}\leq 0.8,\;17\leq x_{3}\leq 28,\;7.3\leq x_{4}\leq 8.3,\;7.8\leq x_{5}\;\leq 8.3,\;2.9\leq x_{6}\leq 3.9,\;\mathrm{and}\;5\leq x_{7}\leq 5.5.$$

The results of implementing the proposed SABO approach and its competing algorithms on the speed reducer design problem are presented in Table 9 and Table 10.

**Figure 8:**
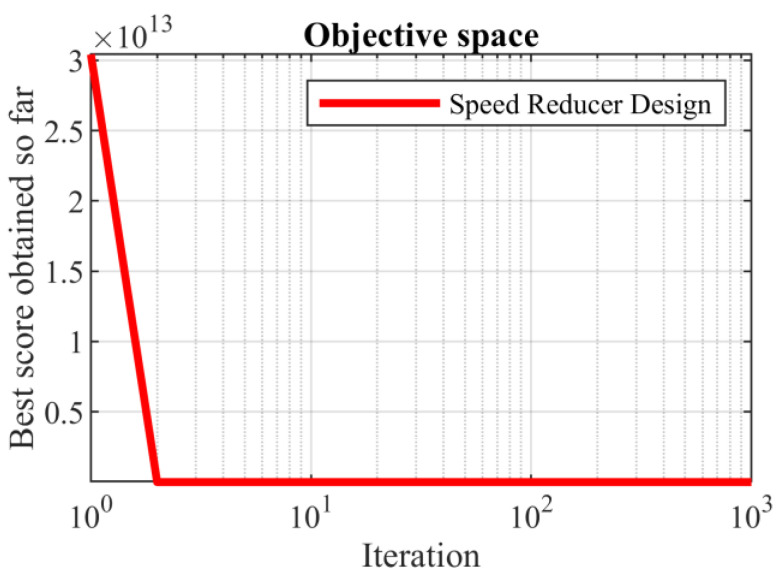
SABO’s performance convergence curve for the speed reducer design.

Based on the obtained results, the SABO provided the optimal solution, with the values of the design variables being equal to (3.5, 0.7, 17, 7.3, 7.8, 3.350214666, and 5.28668323) and the value of the objective function being equal to 2996.348165. What can be concluded from the comparison of the simulation results is that the proposed SABO approach provided better results and a superior performance in dealing with the speed reducer design problem compared to the competing algorithms. The convergence curve of the SABO while achieving the optimal solution for the speed reducer design problem is drawn in Figure 8.

### 5.3. Welded Beam Design

**Figure 9:**
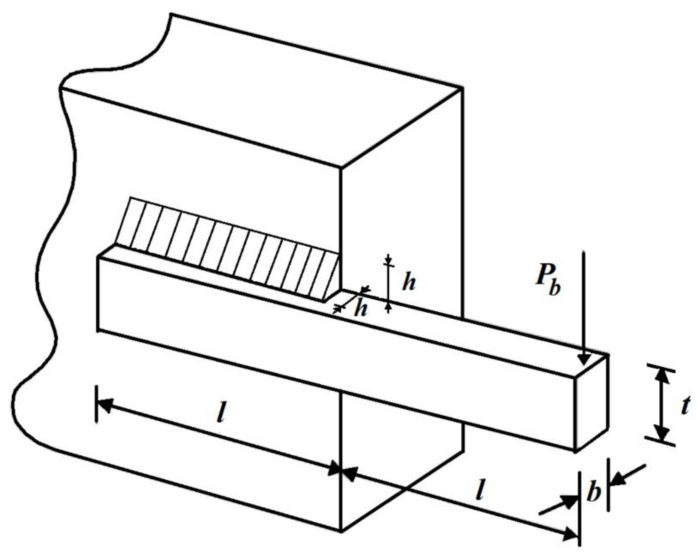
Schematic of the welded beam design.

The design of the welded beam is the subject of optimization by real users to minimize its production costs. The design of the welded beam schematic is shown in Figure 9.

The mathematical model of the welded beam design problem is as follows [32]:

Consider: $X=\left[x_{1},\;x_{2},\;x_{3},\;x_{4}\right]=\left[h,\;l,\;t,\;b\right]$.

Minimize: $f\left(x\right)=1.10471x_{1}^{2}x_{2}+0.04811x_{3}x_{4}\;\left(14.0+x_{2}\right)$.

Subject to:

$$g_{1}\left(x\right)=\tau \left(x\right)-13600\;\leq \;0,\;\;g_{2}\left(x\right)=\sigma \left(x\right)-30000\;\leq \;0,$$

$$g_{3}\left(x\right)=x_{1}-x_{4}\leq \;0,\;\;g_{4}\left(x\right)=0.10471x_{1}^{2}+0.04811x_{3}x_{4}\;\left(14+x_{2}\right)-5.0\;\leq \;0,$$

$$g_{5}\left(x\right)=0.125-x_{1}\leq \;0,\;\;g_{6}\left(x\right)=\delta \;\left(x\right)-0.25\;\leq \;0,$$

$$g_{7}\left(x\right)=6000-p_{c}\;\left(x\right)\leq \;0.$$

where

$$\tau \left(x\right)=\sqrt{{\left(\tau^{\prime }\right)}^{2}+\left(2\tau \tau^{\prime }\right)\frac{x_{2}}{2R}+{\left(\tau \prime \prime \right)}^{2}\;},\;\tau^{\prime }=\frac{6000}{\sqrt{2}x_{1}x_{2}},\;\tau ”=\frac{MR}{J},$$

$$M=6000\left(14+\frac{x_{2}}{2}\right),\;R=\sqrt{\frac{x_{2}^{2}}{4}+{\left(\frac{x_{1}+x_{3}}{2}\right)}^{2}},$$

$$J=2x_{1}x_{2}\sqrt{2}\left(\frac{x_{2}^{2}}{12}+{\left(\frac{x_{1}+x_{3}}{2}\right)}^{2}\right),\;\sigma \left(x\right)=\frac{504000}{x_{4}x_{3}^{2}},$$

$$\delta \;\left(x\right)=\frac{65856000}{\left(30\cdot 10^{6}\right)x_{4}x_{3}^{3}},\;p_{c}\;\left(x\right)=\frac{4.013\left(30\cdot 10^{6}\right)\sqrt{\frac{x_{3}^{2}x_{4}^{6}}{36}}}{196}\left(1-\frac{x_{3}}{28}\sqrt{\frac{30\cdot 10^{6}}{4\left(12\cdot 10^{6}\right)}}\right).$$

with

$$0.1\leq x_{1},\;x_{4}\leq 2\;\mathrm{and}\;0.1\leq x_{2},\;x_{3}\leq 10.$$

The results of using the SABO and its competitor algorithms on the welded beam design problem are reported in Table 11 and Table 12.

**Figure 10:**
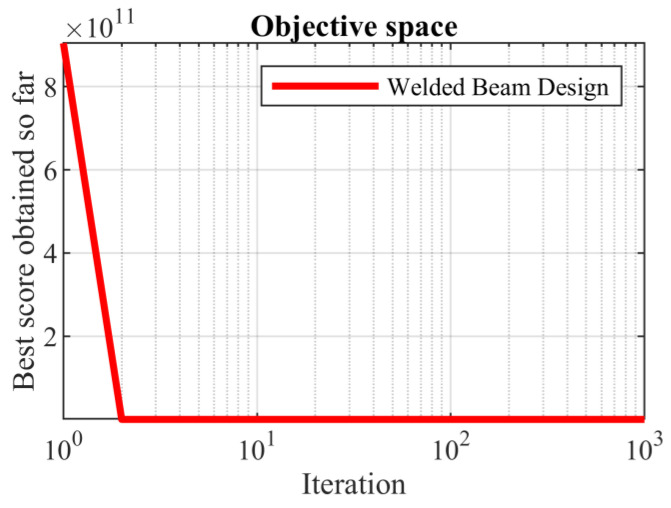
SABO’s performance convergence curve for the welded beam design.

Based on the obtained results, the SABO provided the optimal solution, with the values of the design variables being equal to (0.20572964, 3.470488666, 9.03662391, and 0.20572964) and the value of the objective function being equal to 1.724852309. Comparing these optimization results indicates the superior performance of the SABO over the competing algorithms in optimizing the welded beam design. The SABO convergence curve while providing the solution for the welded beam design problem is drawn in Figure 10.

### 5.4. Tension/Compression Spring Design

**Figure 11:**
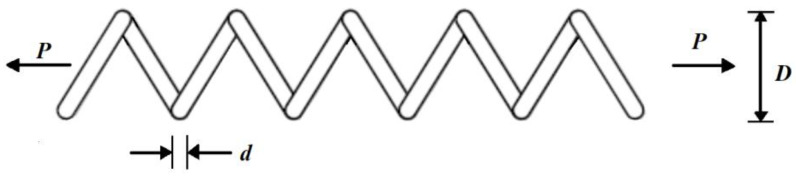
Schematic of the tension/compression spring design.

The tension/compression spring design is an engineering challenge with the aim of minimizing the weight of the tension/compression spring. The tension/compression spring design schematic is shown in Figure 11.

The mathematical model of the tension/compression spring design problem is as follows [32]:

Consider: $X=\left[x_{1},\;x_{2},\;x_{3}\;\right]=\left[d,\;D,\;P\right].$

Minimize: $f\left(x\right)=\left(x_{3}+2\right)x_{2}x_{1}^{2}.$

Subject to:

$$g_{1}\left(x\right)=1-\frac{x_{2}^{3}x_{3}}{71785x_{1}^{4}}\;\leq \;0,\;g_{2}\left(x\right)=\frac{4x_{2}^{2}-x_{1}x_{2}}{12566\left(x_{2}x_{1}^{3}\right)}+\frac{1}{5108x_{1}^{2}}-1\leq \;0,$$

$$g_{3}\left(x\right)=1-\frac{140.45x_{1}}{x_{2}^{2}x_{3}}\leq \;0,\;g_{4}\left(x\right)=\frac{x_{1}+x_{2}}{1.5}-1\;\leq \;0.$$

with

$$0.05\leq x_{1}\leq 2,\;\;0.25\leq x_{2}\leq 1.3\;\mathrm{and}\;2\leq \;x_{3}\leq 15.$$

The results of employing the SABO and the competing algorithms to handle the tension/compression spring design problem are presented in Table 13 and Table 14.

**Figure 12:**
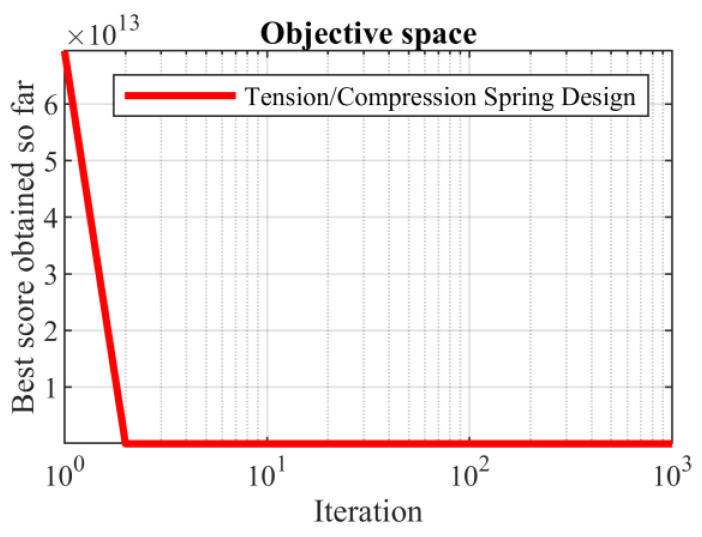
SABO’s performance convergence curve for the tension/compression spring.

Based on the obtained results, the SABO provided the optimal solution, with the values of the design variables being equal to (0.051689061, 0.356717736, and 11.28896595) and the value of the objective function being equal to 0.012665233. What is evident from the analysis of the simulation results is that the SABO was more effective in optimizing the tension/compression spring design than the competing algorithms. The SABO convergence curve in reaching the optimal design for the tension/compression spring problem is drawn in Figure 12.

## 6. Conclusions and Future Works

In this paper, a new metaheuristic algorithm called the Subtraction Average of Searcher Agents (SABO) was designed. The main idea of the design of the SABO was to use mathematical concepts and information on the average differences of searcher agents to update the population of the algorithm. The mathematical modeling of the proposed SABO approach was presented for optimization applications. The SABO’s ability to solve these optimization problems was evaluated for fifty-two standard benchmark functions, including unimodal, high-dimensional, and fixed-dimensional functions, and the CEC 2017 test suite. The optimization results indicated the SABO’s optimal ability to create a balance between exploration and exploitation while scanning the search space to provide suitable solutions for the optimization problems. A total of twelve well-known metaheuristic algorithms were employed for comparison with the proposed SABO approach. Comparing the simulation results showed that the SABO performed better than its competitor algorithms, providing better results for most of the benchmark functions. The implementation of the proposed optimization method on four engineering design problems demonstrated the SABO’s ability to handle these optimization tasks in real-world applications.

With the introduction of the proposed SABO approach, several research avenues are opened for further study. The design of binary and multi-objective versions of the SABO is one of this study’s most special research potentials. Employing the SABO to solve the optimization problems within various sciences and real-world applications is another suggestion for further studies.

## Figures and Tables

**Table 1:** Control parameters values.

Algorithm	Parameter	Value
GA		
	Type	Real coded
	Selection	Roulette wheel (Proportionate)
	Crossover	Whole arithmetic (Probability = 0.8, α∈[−0.5, 1.5])
	Mutation	Gaussian (Probability = 0.05)
PSO		
	Topology	Fully connected
	Cognitive and social constant	(*C*_1_, *C*_2_) = (2, 2)
	Inertia weight	Linear reduction from 0.9 to 0.1
	Velocity limit	10% of dimension range
GSA		
	Alpha, *G*_0_, *R_norm_*, *R_power_*	20, 100, 2, 1
TLBO		
	*T_F_*: teaching factor	*T_F_* = round [(1+rand)]
	random number	*rand* is a random number between [0–1].
GWO		
	Convergence parameter (*a*)	*a*: Linear reduction from 2 to 0.
MVO		
	Wormhole existence probability (WEP)	Min(WEP) = 0.2 and Max(WEP) = 1.
	Exploitation accuracy over the iterations (*p*)	*p* = 6.
WOA		
	Convergence parameter (*a*)	*a*: Linear reduction from 2 to 0.
	*r* is a random vector in [0–1]	
	*l* is a random number in [−1,1].	
TSA		
	P_min_ and P_max_	1, 4
	*c*1, *c*2, *c*3	Random numbers lie in the range of [0–1].
MPA		
	Constant number	*p* = 0.5
	Random vector	*R* is a vector of uniform random numbers in [0, 1].
	Fish Aggregating Devices (*FADs*)	*FADs* = 0.2
	Binary vector	*U* = 0 or 1
RSA		
	Sensitive parameter	β=0.01
	Sensitive parameter	α=0.1
	Evolutionary Sense (ES)	ES: randomly decreasing values between 2 and −2
AVOA		
	L_1_, L_2_	0.8, 0.2
	w	2.5
	P_1_, P_2_, P_3_	0.6, 0.4, 0.6
WSO		
	F_min_ and F_max_	0.07, 0.75
	*τ*, *a_o_*, *a*_1_, *a*_2_	4.125, 6.25, 100, 0.0005

**Table 2:** Optimization results of unimodal functions (F1–F8).

		SABO	WSO	AVOA	RSA	MPA	TSA	WOA	MVO	GWO	TLBO	GSA	PSO	GA
F1	Mean	0	94.68556	0	0	6.88 × 10^−50^	6.07 × 10^−47^	2.4 × 10^−155^	0.144595	3.87 × 10^−58^	8.14 × 10^−75^	9.33 × 10^−17^	0.022702	30.50201
	Best	0	17.42507	0	0	2.13 × 10^−51^	1.01 × 10^−50^	1.4 × 10^−167^	0.071226	7.24 × 10^−61^	5.32 × 10^−77^	4.88 × 10^−17^	4.43 × 10^−6^	17.92696
	Worst	0	439.1736	0	0	5.24 × 10^−49^	6.22 × 10^−46^	2.1 × 10^−154^	0.269577	6.86 × 10^−57^	1.07 × 10^−73^	1.92 × 10^−16^	0.158149	56.92799
	Std	0	101.7604	0	0	1.26 × 10^−49^	1.64 × 10^−46^	6.1 × 10^−155^	0.05687	1.52 × 10^−57^	2.34 × 10^−74^	3.76 × 10^−17^	0.046942	10.46286
	Median	0	51.91934	0	0	1.97 × 10^−50^	4.04 × 10^−48^	3.7 × 10^−158^	0.122317	1.34 × 10^−59^	1.27 × 10^−75^	8.64 × 10^−17^	0.001314	28.19897
	Rank	1	11	1	1	5	6	2	9	4	3	7	8	10
F2	Mean	0	1.575136	1.2 × 10^−266^	0	3 × 10^−28^	1.11 × 10^−28^	5.7 × 10^−103^	0.26717	7.97 × 10^−35^	6.09 × 10^−39^	5.22 × 10^−8^	0.731055	2.788395
	Best	0	0.609154	2.3 × 10^−301^	0	3.21 × 10^−31^	1.02 × 10^−30^	3.8 × 10^−114^	0.189084	1.45 × 10^−35^	3.25 × 10^−40^	3.41 × 10^−8^	0.089719	1.745356
	Worst	0	4.873582	2.5 × 10^−265^	0	1.5 × 10^−27^	5.72 × 10^−28^	5.3 × 10^−102^	0.457641	2.54 × 10^−34^	3.59 × 10^−38^	7.3 × 10^−8^	1.908597	3.806556
	Std	0	1.088388	0	0	4.14 × 10^−28^	1.6 × 10^−28^	1.5 × 10^−102^	0.075891	6.78 × 10^−35^	9.92 × 10^−39^	1.14 × 10^−8^	0.534508	0.544788
	Median	0	1.202514	7.1 × 10^−287^	0	1.17 × 10^−28^	3.98 × 10^−29^	9 × 10^−108^	0.253065	6.46 × 10^−35^	2.45 × 10^−39^	5.16 × 10^−8^	0.743555	2.741555
	Rank	1	11	2	1	7	6	3	9	5	4	8	10	12
F3	Mean	0	1806.78	0	0	5.38 × 10^−12^	1.31 × 10^−12^	21,771.78	14.05216	4.07 × 10^−15^	1.87 × 10^−25^	434.0065	643.4302	2168.983
	Best	0	687.9998	0	0	2.9 × 10^−25^	1.4 × 10^−17^	804.8555	5.810656	3.22 × 10^−19^	1.87 × 10^−29^	235.952	36.45082	1424.187
	Worst	0	4051.23	0	0	7.54 × 10^−11^	1.89 × 10^−11^	39,997.34	29.19354	4.98 × 10^−14^	1.97 × 10^−24^	905.2518	5210.771	3458.935
	Std	0	827.0454	0	0	1.71 × 10^−11^	4.22 × 10^−12^	10,690.48	6.15891	1.13 × 10^−14^	4.64 × 10^−25^	157.388	1118.449	639.6914
	Median	0	1619.412	0	0	2.19 × 10^−13^	2.52 × 10^−14^	23,134.65	12.09381	2.84 × 10^−16^	1.35 × 10^−26^	418.4298	284.912	2100.7
	Rank	1	9	1	1	5	4	11	6	3	2	7	8	10
F4	Mean	0	17.76181	2 × 10^−263^	0	4.29 × 10^−19^	0.004673	44.49878	0.52347	1.32 × 10^−14^	3.14 × 10^−30^	0.763785	6.431779	2.829395
	Best	0	11.90369	0	0	9.98 × 10^−20^	3.08 × 10^−5^	3.549529	0.290684	3.65 × 10^−16^	1.45 × 10^−31^	1.23 × 10^−8^	3.435068	2.216469
	Worst	0	24.61971	3.6 × 10^−262^	0	1.39 × 10^−18^	0.026002	92.11975	0.898058	5.88 × 10^−14^	1.33 × 10^−29^	4.299889	14.35043	3.992738
	Std	0	3.607365	0	0	3.25 × 10^−19^	0.006495	30.23659	0.163563	1.68 × 10^−14^	3.51 × 10^−30^	1.049333	2.439277	0.466936
	Median	0	16.86055	2.9 × 10^−282^	0	3.93 × 10^−19^	0.003083	40.15902	0.5337	7.33 × 10^−15^	1.86 × 10^−30^	0.402748	6.046926	2.783478
	Rank	1	11	2	1	4	6	12	7	5	3	8	10	9
F5	Mean	0.197101	11,081.32	1.87205	11.53391	23.55614	28.42991	27.20948	392.7405	26.99155	26.88666	26.39314	84.19977	595.3854
	Best	0.003263	455.9772	1.58657	8.2 × 10^−29^	22.95066	26.0171	26.53351	24.75643	26.01628	25.61541	25.88273	11.41045	228.808
	Worst	0.81947	44,603	2.13145	28.99015	24.83591	29.21115	28.51838	2433.592	27.94714	28.74413	27.72119	178.5254	2257.058
	Std	0.215288	14,373.09	1.542075	14.49384	0.436322	0.779248	0.474719	735.2203	0.5881	0.964054	0.426365	44.9786	424.9867
	Median	0.111639	2840.274	1.47245	1.08 × 10^−28^	23.42466	28.82636	27.0195	30.39723	27.11413	26.45399	26.28991	87.48235	475.573
	Rank	1	13	2	3	4	9	8	11	7	6	5	10	12
F6	Mean	0	119.0172	6.52 × 10^−8^	6.319044	1.77 × 10^−9^	3.683762	0.086202	0.153294	0.636332	1.116967	1.07 × 10^−16^	0.082637	34.14746
	Best	0	15.05144	4.73 × 10^−9^	3.88797	6.98 × 10^−10^	2.821592	0.002679	0.092788	0.249403	0.487407	4.96 × 10^−17^	5.23 × 10^−5^	15.61244
	Worst	0	618.6501	2.46 × 10^−7^	7.452363	4.45 × 10^−9^	4.79066	0.429712	0.2525	1.258956	1.907377	1.92 × 10^−16^	1.549095	62.76702
	Std	0	131.4271	5.56 × 10^−8^	1.201026	9.43 × 10^−10^	0.543926	0.110313	0.039617	0.309366	0.409251	3.8 × 10^−17^	0.345263	13.54999
	Median	0	78.07582	5.69 × 10^−8^	6.999263	1.41 × 10^−9^	3.565372	0.03592	0.148684	0.501812	1.043402	9.84 × 10^−17^	0.002623	31.68218
	Rank	1	13	4	11	3	10	6	7	8	9	2	5	12
F7	Mean	2.38 × 10^−6^	4.93 × 10^−5^	5.44 × 10^−5^	4.88 × 10^−5^	0.00056	0.005326	0.002244	0.011754	0.00091	0.001542	0.059762	0.168645	0.010589
	Best	1.74 × 10^−7^	4.44 × 10^−7^	2.41 × 10^−7^	3.72 × 10^−6^	0.000225	0.002506	1.76 × 10^−5^	0.005824	0.000117	0.000261	0.024813	0.074626	0.003032
	Worst	7.52 × 10^−6^	0.000128	0.000152	0.000226	0.001089	0.016372	0.010815	0.020623	0.00202	0.003007	0.102681	0.293086	0.021939
	Std	1.98 × 10^−6^	3.94 × 10^−5^	5.06 × 10^−5^	5.09 × 10^−5^	0.000258	0.003333	0.002743	0.004162	0.000569	0.000772	0.021243	0.060668	0.004819
	Median	1.63 × 10^−6^	5.39 × 10^−5^	3.64 × 10^−5^	3.21 × 10^−5^	0.00048	0.004299	0.001254	0.010535	0.000758	0.001479	0.056525	0.152506	0.010178
	Rank	1	3	4	2	5	9	8	11	6	7	12	13	10
Sum rank		7	71	16	20	33	50	50	60	38	34	49	64	75
Mean rank		1	10.14286	2.285714	2.857143	4.714286	7.142857	7.142857	8.571429	5.428571	4.857143	7	9.142857	10.71429
Total rank		1	11	2	3	4	8	8	9	6	5	7	10	12

**Table 3:** Optimization results of high-dimensional multimodal functions (F8–F13).

		SABO	WSO	AVOA	RSA	MPA	TSA	WOA	MVO	GWO	TLBO	GSA	PSO	GA
F8	Mean	−12,563.1	−7037.55	−12,433.2	−5458.28	−9865.61	−5913.41	−11,247.2	−7742.72	−6220.73	−5521.56	−2689.18	−6500.96	−8421.5
	Best	−12,569.5	−8624.3	−12,569.5	−5656.04	−10,653.2	−6776.47	−12,569.2	−9182.08	−8101.01	−6451.23	−3269.84	−7862.41	−9681.18
	Worst	−12,447.1	−5826.04	−11,896.8	−4124.78	−9067.64	−4968.21	−6824.1	−6283.06	−3450.14	−4631.24	−2140.43	−4751.67	−7028.99
	Std	27.32588	840.3862	197.7043	345.5191	478.9808	494.6759	1769.136	677.1584	896.6881	562.8207	341.7721	885.9265	641.2242
	Median	−12,569.5	−7012.04	−12,569.5	−5531.08	−9792.7	−5881.29	−12,081.1	−7915.87	−6226.44	−5625.4	−2654.18	−6783.5	−8399.11
	Rank	1	7	2	12	4	10	3	6	9	11	13	8	5
F9	Mean	0	30.53863	0	0	0	190.2096	0	104.0543	0.297985	0	25.07295	60.31323	54.68123
	Best	0	15.22149	0	0	0	92.78168	0	43.82732	0	0	13.92943	29.84883	23.23239
	Worst	0	68.24684	0	0	0	273.0471	0	152.2773	5.959691	0	41.78816	113.4265	76.90086
	Std	0	11.93438	0	0	0	40.82636	0	28.71635	1.332627	0	6.256114	21.62223	13.80758
	Median	0	30.62966	0	0	0	189.0894	0	99.60579	0	0	23.879	56.24334	52.61443
	Rank	1	4	1	1	1	8	1	7	2	1	3	6	5
F10	Mean	8.88 × 10^−16^	4.901082	8.88 × 10^−16^	8.88 × 10^−16^	4.44 × 10^−15^	1.452865	4.26 × 10^−15^	0.451449	1.6 × 10^−14^	4.26 × 10^−15^	8.12 × 10^−9^	2.739329	3.5751
	Best	8.88 × 10^−16^	3.530049	8.88 × 10^−16^	8.88 × 10^−16^	4.44 × 10^−15^	7.99 × 10^−15^	8.88 × 10^−16^	0.078241	1.15 × 10^−14^	8.88 × 10^−16^	5.45 × 10^−9^	1.778035	2.881962
	Worst	8.88 × 10^−16^	6.874831	8.88 × 10^−16^	8.88 × 10^−16^	4.44 × 10^−15^	3.447315	7.99 × 10^−15^	1.799202	2.22 × 10^−14^	4.44 × 10^−15^	1.23 × 10^−8^	4.38263	4.641967
	Std	0	0.939579	0	0	0	1.654267	2.44 × 10^−15^	0.574717	2.79 × 10^−15^	7.94 × 10^−16^	1.67 × 10^−9^	0.715238	0.396644
	Median	8.88 × 10^−16^	4.668923	8.88 × 10^−16^	8.88 × 10^−16^	4.44 × 10^−15^	2.22 × 10^−14^	4.44 × 10^−15^	0.131373	1.51 × 10^−14^	4.44 × 10^−15^	7.81 × 10^−9^	2.604421	3.62958
	Rank	1	10	1	1	3	7	2	6	4	2	5	8	9
F11	Mean	0	1.70897	0	0	0	0.008334	0	0.412213	0.000451	0	8.687165	0.156717	1.473471
	Best	0	1.076151	0	0	0	0	0	0.191432	0	0	3.135355	0.001467	1.288095
	Worst	0	5.872952	0	0	0	0.067031	0	0.535573	0.009011	0	15.71589	1.662839	1.725859
	Std	0	1.0671	0	0	0	0.01536	0	0.099926	0.002015	0	3.751821	0.360388	0.123868
	Median	0	1.425853	0	0	0	0	0	0.430656	0	0	7.888906	0.060533	1.447709
	Rank	1	7	1	1	1	3	1	5	2	1	8	4	6
F12	Mean	2.63 × 10^−33^	34.6973	2.86 × 10^−9^	1.314643	2.08 × 10^−10^	6.293599	0.00738	1.251754	0.035525	0.08398	0.187602	1.583141	0.274894
	Best	2.13 × 10^−34^	0.938867	7.56 × 10^−10^	0.720132	6.03 × 10^−11^	0.216628	0.000964	0.001016	0.012978	0.035054	5.91 × 10^−19^	0.074149	0.060841
	Worst	5.73 × 10^−33^	597.7173	5.14 × 10^−9^	1.629701	4.75 × 10^−10^	17.71439	0.033587	6.169218	0.07355	0.170805	0.634329	5.095104	0.650842
	Std	1.57 × 10^−33^	132.7047	1.34 × 10^−9^	0.330125	9.3 × 10^−11^	4.26837	0.007596	1.62632	0.018405	0.032215	0.20951	1.251155	0.138648
	Median	2.62 × 10^−33^	3.696076	2.7 × 10^−9^	1.525877	1.93 × 10^−10^	6.01544	0.005285	0.807143	0.029024	0.082474	0.155493	1.381006	0.264424
	Rank	1	13	3	10	2	12	4	9	5	6	7	11	8
F13	Mean	6.7 × 10^−32^	4239.934	1.43 × 10^−8^	0.355	0.000567	2.81567	0.275667	0.02976	0.495453	1.030391	0.007691	4.690251	2.707835
	Best	1.14 × 10^−34^	12.39891	1.5 × 10^−9^	6.53 × 10^−32^	8.3 × 10^−10^	2.029692	0.032988	0.009002	2.27 × 10^−5^	0.529644	5.94 × 10^−18^	0.04709	1.291959
	Worst	4.34 × 10^−31^	17,963.65	3.86 × 10^−8^	2.9	0.011347	3.832826	0.781805	0.079707	0.852264	1.626638	0.098883	14.57619	3.940231
	Std	1.2 × 10^−31^	7400.496	1.17 × 10^−8^	0.849443	0.002537	0.479843	0.209069	0.018477	0.219041	0.29138	0.022003	4.549049	0.754476
	Median	3.38 × 10^−32^	55.13453	1.1 × 10^−8^	9.24 × 10^−32^	2.33 × 10^−9^	2.823652	0.233889	0.023238	0.569144	1.031507	1.1 × 10^−17^	3.216389	2.867222
	Rank	1	13	2	7	3	11	6	5	8	9	4	12	10
Sum rank		6	54	10	32	14	51	17	38	30	30	40	49	43
Mean rank		1	9	1.666667	5.333333	2.333333	8.5	2.833333	6.333333	5	5	6.666667	8.166667	7.166667
Total rank		1	12	2	6	3	11	4	7	5	5	8	10	9

**Table 4:** Optimization results of high-dimensional multimodal functions (F14–F23).

		SABO	WSO	AVOA	RSA	MPA	TSA	WOA	MVO	GWO	TLBO	GSA	PSO	GA
F14	Mean	0.998004	1.146516	1.295817	3.070575	0.998004	9.656354	1.783898	0.998004	4.423582	1.09721	3.999176	3.9306	1.048667
	Best	0.998004	0.998004	0.998004	0.998031	0.998004	0.998004	0.998004	0.998004	0.998004	0.998004	0.998004	0.998004	0.998004
	Worst	0.998004	3.96825	2.982105	10.76318	0.998004	17.37441	10.76318	0.998004	10.76318	2.982105	8.849513	15.50382	1.992037
	Std	7.2 × 10^−17^	0.664167	0.651946	2.170562	7.2 × 10^−17^	5.167271	2.233902	3.74 × 10^−12^	4.335554	0.443658	2.698996	4.397024	0.222066
	Median	0.998004	0.998004	0.998004	2.982105	0.998004	12.67051	0.998004	0.998004	2.982105	0.998004	3.146201	2.487068	0.998004
	Rank	1	5	6	8	1	12	7	2	11	4	10	9	3
F15	Mean	0.000307	0.000308	0.000341	0.001134	0.000712	0.008416	0.00061	0.004474	0.004475	0.003436	0.002272	0.005546	0.015388
	Best	0.000307	0.000307	0.000308	0.000538	0.000307	0.000308	0.00031	0.000348	0.000307	0.000308	0.001538	0.000307	0.000782
	Worst	0.000307	0.000316	0.000527	0.00212	0.002252	0.056621	0.001502	0.056543	0.020363	0.020364	0.004034	0.056543	0.066917
	Std	2.29 × 10^−19^	1.87 × 10^−6^	6.5 × 10^−5^	0.000451	0.000665	0.014443	0.000307	0.013022	0.00816	0.007301	0.000649	0.013444	0.016221
	Median	0.000307	0.000307	0.000309	0.00099	0.000314	0.000627	0.000573	0.00062	0.000308	0.000317	0.00208	0.000444	0.014273
	Rank	1	2	3	6	5	12	4	9	10	8	7	11	13
F16	Mean	−1.03163	−1.03163	−1.03163	−1.02933	−1.03163	−1.02688	−1.03163	−1.03163	−1.03163	−1.03163	−1.03163	−1.03163	−1.03163
	Best	−1.03163	−1.03163	−1.03163	−1.03162	−1.03163	−1.03163	−1.03163	−1.03163	−1.03163	−1.03163	−1.03163	−1.03163	−1.03163
	Worst	−1.03163	−1.03163	−1.03163	−1	−1.03163	−1	−1.03163	−1.03163	−1.03163	−1.03162	−1.03163	−1.03163	−1.03161
	Std	2.1 × 10^−16^	8.4 × 10^−8^	8.82 × 10^−17^	0.006971	1.91 × 10^−16^	0.011587	1.17 × 10^−10^	4.03 × 10^−8^	5.64 × 10^−9^	1.33 × 10^−6^	1.53 × 10^−16^	8.82 × 10^−17^	4.78 × 10^−6^
	Median	−1.03163	−1.03163	−1.03163	−1.0312	−1.03163	−1.03163	−1.03163	−1.03163	−1.03163	−1.03163	−1.03163	−1.03163	−1.03163
	Rank	1	4	1	8	1	9	2	5	3	6	1	1	7
F17	Mean	0.397887	0.397895	0.397887	0.409183	0.397887	0.39792	0.397888	0.397887	0.397888	0.400047	0.397887	0.52702	0.466023
	Best	0.397887	0.397887	0.397887	0.397962	0.397887	0.397888	0.397887	0.397887	0.397887	0.3979	0.397887	0.397887	0.397887
	Worst	0.397887	0.398048	0.397887	0.498535	0.397887	0.398075	0.397892	0.397888	0.397891	0.437578	0.397887	1.130918	1.75218
	Std	0	3.59 × 10^−5^	3.98 × 10^−16^	0.023274	0	4.3 × 10^−5^	1.18 × 10^−6^	1.05 × 10^−7^	8.45 × 10^−7^	0.008836	0	0.254527	0.302731
	Median	0.397887	0.397887	0.397887	0.401719	0.397887	0.397907	0.397887	0.397887	0.397888	0.397997	0.397887	0.397887	0.397905
	Rank	1	6	2	9	1	7	5	3	4	8	1	11	10
F18	Mean	3	3	3.000003	5.742379	3	12.45003	3.000021	3	3.000012	3.000001	3	3	7.302903
	Best	3	3	3	3	3	3.000001	3	3	3.000001	3	3	3	3
	Worst	3	3	3.000028	30.75151	3	84.00011	3.000169	3.000001	3.000038	3.000006	3	3	34.94955
	Std	9.11 × 10^−16^	4.2 × 10^−16^	6.42 × 10^−6^	8.441297	1.55 × 10^−15^	25.19918	3.78 × 10^−5^	3.94 × 10^−7^	9.8 × 10^−6^	1.56 × 10^−6^	3.02 × 10^−15^	2.58 × 10^−15^	10.54375
	Median	3	3	3.000001	3.000014	3	3.00001	3.00001	3	3.00001	3	3	3	3.00117
	Rank	1	1	7	10	2	12	9	5	8	6	4	3	11
F19	Mean	−3.86278	−3.86278	−3.86278	−3.82685	−3.86278	−3.86274	−3.86058	−3.86278	−3.86072	−3.86047	−3.86278	−3.86278	−3.86262
	Best	−3.86278	−3.86278	−3.86278	−3.86048	−3.86278	−3.86278	−3.86278	−3.86278	−3.86278	−3.86273	−3.86278	−3.86278	−3.86278
	Worst	−3.86278	−3.86278	−3.86278	−3.73516	−3.86278	−3.86264	−3.85204	−3.86278	−3.8549	−3.85483	−3.86278	−3.86278	−3.86183
	Std	2.28 × 10^−15^	2.28 × 10^−15^	3.89 × 10^−13^	0.038307	2.13 × 10^−15^	3.9 × 10^−5^	0.002904	1.87 × 10^−7^	0.003273	0.003341	1.9 × 10^−15^	2.06 × 10^−15^	0.000295
	Median	−3.86278	−3.86278	−3.86278	−3.8444	−3.86278	−3.86276	−3.86171	−3.86278	−3.86277	−3.86245	−3.86278	−3.86278	−3.86278
	Rank	1	1	2	9	1	4	7	3	6	8	1	1	5
F20	Mean	−3.322	−3.29813	−3.26819	−2.87036	−3.28036	−3.26468	−3.23382	−3.25652	−3.25038	−3.21772	−3.322	−3.29494	−3.2283
	Best	−3.322	−3.322	−3.322	−3.07689	−3.322	−3.32164	−3.32198	−3.32199	−3.32199	−3.31795	−3.322	−3.322	−3.32163
	Worst	−3.322	−3.20308	−3.1971	−2.48983	−3.2031	−3.1574	−3.0863	−3.2028	−3.085	−3.02017	−3.322	−3.13764	−2.99723
	Std	4.32 × 10^−16^	0.048752	0.061042	0.155393	0.058168	0.064086	0.09432	0.060761	0.095649	0.079637	4.08 × 10^−16^	0.057012	0.078203
	Median	−3.322	−3.322	−3.322	−2.89614	−3.32199	−3.3194	−3.25911	−3.20305	−3.32199	−3.19712	−3.322	−3.322	−3.23661
	Rank	1	2	5	12	4	6	9	7	8	11	1	3	10
F21	Mean	−10.1532	−9.77968	−10.1532	−5.0552	−10.1532	−6.90572	−8.24533	−7.99956	−9.64755	−5.86981	−5.69611	−7.15161	−6.26023
	Best	−10.1532	−10.1532	−10.1532	−5.0552	−10.1532	−10.0952	−10.1532	−10.1532	−10.1532	−8.27119	−10.1532	−10.1532	−9.73855
	Worst	−10.1532	−2.68286	−10.1532	−5.0552	−10.1532	−2.61113	−2.6301	−2.63047	−5.10027	−4.17485	−2.63047	−2.63047	−2.38578
	Std	2.61 × 10^−15^	1.670419	2.03 × 10^−14^	3.1 × 10^−7^	1.03 × 10^−7^	3.54775	2.711952	2.756513	1.555113	1.573889	3.51533	3.494447	2.711083
	Median	−10.1532	−10.1532	−10.1532	−5.0552	−10.1532	−9.86587	−10.1506	−10.1531	−10.1528	−4.88288	−3.46205	−10.1532	−7.06069
	Rank	1	4	2	13	3	9	6	7	5	11	12	8	10
F22	Mean	−10.4029	−9.73508	−10.4029	−5.08767	−10.4029	−8.2408	−7.7929	−8.9621	−10.1367	−7.82384	−10.4029	−5.31476	−7.37187
	Best	−10.4029	−10.4029	−10.4029	−5.08767	−10.4029	−10.3533	−10.4029	−10.4029	−10.4028	−10.0684	−10.4029	−10.4029	−9.9828
	Worst	−10.4029	−3.7243	−10.4029	−5.08767	−10.4029	−1.83234	−2.76573	−2.76589	−5.08766	−3.63254	−10.4029	−2.75193	−2.67682
	Std	3.65 × 10^−15^	2.055642	3.13 × 10^−14^	6.98 × 10^−7^	3.62 × 10^−15^	3.482703	2.946435	2.605072	1.18842	1.928487	2.79 × 10^−15^	3.465308	1.916626
	Median	−10.4029	−10.4029	−10.4029	−5.08767	−10.4029	−10.1656	−10.0872	−10.4029	−10.4025	−8.44314	−10.4029	−3.2451	−7.86313
	Rank	1	5	3	12	1	7	9	6	4	8	2	11	10
F23	Mean	−10.5364	−10.1307	−10.5364	−5.12847	−10.5364	−8.08868	−8.25778	−10.266	−10.5361	−7.60728	−10.5364	−5.56226	−6.36016
	Best	−10.5364	−10.5364	−10.5364	−5.12848	−10.5364	−10.4974	−10.5363	−10.5364	−10.5364	−10.3064	−10.5364	−10.5364	−10.1845
	Worst	−10.5364	−2.42173	−10.5364	−5.12847	−10.5364	−2.41711	−1.67653	−5.12846	−10.5357	−3.91631	−10.5364	−2.42734	−2.38229
	Std	2.51 × 10^−15^	1.814497	3.97 × 10^−15^	1.53 × 10^−6^	2.85 × 10^−15^	3.633979	3.217511	1.209244	0.000146	1.800721	1.63 × 10^−15^	3.772667	2.608634
	Median	−10.5364	−10.5364	−10.5364	−5.12847	−10.5364	−10.3713	−10.5338	−10.5364	−10.5361	−8.05319	−10.5364	−3.35328	−6.88826
	Rank	1	5	2	11	1	7	6	4	3	8	1	10	9
Sum rank		10	35	33	98	20	85	64	51	62	78	40	68	88
Mean rank		1	3.5	3.3	9.8	2	8.5	6.4	5.1	6.2	7.8	4	6.8	8.8
Total rank		1	4	3	13	2	11	8	6	7	10	5	9	12

**Table 5:** Optimization results of the CEC 2017 test suite.

		SABO	WSO	AVOA	RSA	MPA	TSA	WOA	MVO	GWO	TLBO	GSA	PSO	GA
C17-F1	Mean	100	7046.882	1840.86	9.82 × 10^9^	10,040.45	1.34 × 10^9^	7,369,265	11,894.28	19,142.49	1.59 × 10^8^	332.224	3380.706	20,223,617
	Best	100	349.4302	761.674	7.37 × 10^9^	2897.088	11,782,641	3,382,781	6418.842	11,813.99	70,630,075	110.2765	365.6412	6,742,684
	Worst	100	12,596.3	3920.943	1.28 × 10^10^	13,813.56	3.84 × 10^9^	10,346,872	16,013.61	28,455.49	3.83 × 10^8^	754.7187	10,023.59	37,058,367
	Std	1.76 × 10^−5^	6377.11	1455.264	2.72 × 10^9^	4862.963	1.71 × 10^9^	3,477,153	4126.177	7771.845	1.5 × 10^8^	293.2302	4472.088	12,528,147
	Median	100	7620.896	1340.412	9.56 × 10^9^	11,725.58	7.54 × 10^8^	7,873,703	12,572.34	18,150.24	90,563,366	231.9504	1566.796	18,546,709
	Rank	1	5	3	13	6	12	9	7	8	11	2	4	10
C17-F3	Mean	300	353.4634	336.7463	11,593.82	303.0258	11,561.85	1014.038	303.025	3214.45	762.0832	11,528.91	303	15,890.56
	Best	300	305.6803	303.001	7495.814	303.0155	7333.238	506.675	303.0096	618.2583	487.416	9410.586	303	4664.339
	Worst	300	398.7643	379.2247	15,992.88	303.034	15,496.53	1772.56	303.0438	7596.973	941.5065	13,035.42	303	25,128.63
	Std	5.43 × 10^−11^	51.21056	31.53062	4721.028	0.008085	3337.066	557.707	0.014236	3317.442	199.0619	1570.355	4.64 × 10^−14^	10,690.16
	Median	300	354.7045	332.3797	11,443.29	303.0267	11,708.81	888.4577	303.0233	2321.285	809.7051	11,834.82	303	16,884.64
	Rank	1	6	5	12	4	11	8	3	9	7	10	2	13
C17-F4	Mean	400.002	411.7815	422.7311	890.6229	407.5184	588.2896	435.164	408.4423	427.4762	413.8849	410.3218	425.8947	419.8654
	Best	400	404.0126	405.1815	592.3616	406.3748	410.9766	410.9211	407.3072	411.3067	413.039	409.1493	404.1139	416.5881
	Worst	400.008	428.8649	473.5286	1532.186	408.0158	956.8654	461.0965	409.526	475.1348	414.4191	410.867	479.8561	423.8752
	Std	0.004024	11.75615	33.87191	431.9196	0.767374	248.7957	27.51261	0.907625	31.77317	0.591742	0.789237	36.34511	3.18921
	Median	400	407.1243	406.1071	718.9722	407.8415	492.6581	434.3191	408.4681	411.7316	414.0409	410.6354	409.8044	419.4992
	Rank	1	5	8	13	2	12	11	3	10	6	4	9	7
C17-F5	Mean	510.1638	524.345	547.7084	585.8799	516.3361	577.0081	543.3966	529.3387	523.0358	541.9821	558.7547	535.2866	535.4083
	Best	507.9597	515.0492	526.103	566.9905	512.0446	539.5294	519.6511	520.078	515.4188	536.0268	545.1657	517.0589	530.3026
	Worst	513.7912	534.1427	569.3135	597.8354	523.1164	599.001	567.8895	548.081	533.9026	545.8445	572.3282	561.2747	541.6999
	Std	2.551081	8.813038	17.75945	13.40493	4.81413	26.23584	20.0436	12.73497	8.200079	4.315597	12.68207	20.42117	5.160091
	Median	509.4521	524.094	547.7084	589.3468	515.0917	584.7509	543.0228	524.5978	521.411	543.0286	558.7624	531.4064	534.8153
	Rank	1	4	10	13	2	12	9	5	3	8	11	6	7
C17-F6	Mean	600.0003	606.5662	621.0782	650.7423	609.1735	633.9321	646.477	606.5379	607.6657	613.5003	624.6064	614.1202	617.2135
	Best	600.0001	606.0005	611.45	647.7003	607.6625	618.2979	641.2523	606.2218	607.1218	611.2009	615.1481	607.4805	613.5467
	Worst	600.0004	608.0621	641.9305	654.2638	610.8793	650.6244	657.2451	607.0517	609.1673	617.0857	636.0319	627.0489	621.8526
	Std	0.000133	1.001765	14.07778	2.920617	1.432896	13.49693	7.511607	0.38974	1.001744	2.682933	8.726274	8.876072	3.681783
	Median	600.0004	606.1011	615.4663	650.5025	609.0762	633.4031	643.7053	606.4391	607.1869	612.8573	623.6228	610.9757	616.7274
	Rank	1	3	9	13	5	11	12	2	4	6	10	7	8
C17-F7	Mean	720.073	734.6061	761.3859	807.9573	722.6878	832.3335	780.3394	729.1437	737.4493	762.9736	723.9356	741.8665	746.3812
	Best	715.4835	724.1491	735.2345	798.2316	721.6198	802.1454	758.5209	718.6366	729.0264	758.0501	718.5417	733.9806	735.012
	Worst	724.3538	744.5125	809.0966	818.5406	724.5213	871.8311	810.1066	735.7483	757.2095	771.8916	733.1719	754.5434	751.4285
	Std	3.767197	8.718046	32.71951	8.520126	1.302941	28.97113	25.43976	7.630846	13.25034	6.207461	6.432453	9.363563	7.701444
	Median	720.2273	734.8814	750.6063	807.5285	722.305	827.6787	776.3651	731.0949	731.7806	760.9763	722.0144	739.471	749.5423
	Rank	1	5	9	12	2	13	11	4	6	10	3	7	8
C17-F8	Mean	810.4471	816.5418	839.1887	862.826	817.8294	842.4619	847.5424	835.8918	823.7197	849.1191	831.6153	832.7817	826.2435
	Best	807.9597	813.0245	830.2544	848.7521	813.055	820.5764	834.07	817.0478	818.4748	841.6045	824.0785	825.0834	821.9262
	Worst	812.9345	820.059	846.1864	869.0084	821.0988	864.6593	862.7255	864.2865	830.5745	857.9026	840.157	839.7978	834.8115
	Std	2.071168	2.900933	8.233535	9.572994	3.420317	18.78867	11.74116	20.07474	5.330818	8.339208	7.762303	7.284997	5.810643
	Median	810.4471	816.5418	840.157	866.7719	818.5819	842.306	846.6871	831.1164	822.9147	848.4846	831.1129	833.1227	824.1182
	Rank	1	2	9	13	3	10	11	8	4	12	6	7	5
C17-F9	Mean	900	967.6049	1195.105	1536.685	909.5698	1285.153	1156.894	909.1165	909.5976	921.9334	909	913.6391	914.5897
	Best	900	915.8816	1042.267	1391.599	909.0062	940.4326	1015.748	909.0008	909.0575	916.909	909	909.9834	912.06
	Worst	900	1067.154	1401.239	1802.164	910.3272	1718.309	1474.688	909.4606	909.9263	930.8768	909	922.4721	918.9278
	Std	6.63 × 10^−8^	71.14703	150.341	186.13	0.668621	339.4266	213.3433	0.22941	0.37996	6.138603	0	5.963657	3.106199
	Median	900	943.6919	1168.458	1476.489	909.4729	1240.936	1068.57	909.0023	909.7033	919.974	909	911.0504	913.6856
	Rank	1	9	11	13	4	12	10	3	5	8	2	6	7
C17-F10	Mean	1332.824	1480.565	1983.322	2488.599	1425.781	2408.738	2489.529	1812.192	1551.249	2278.76	2590.355	2033.726	1784.313
	Best	1148.146	1252.93	1547.347	2301.942	1233.409	2216.257	2144.702	1622.704	1424.405	1855.109	2170.808	1615.933	1457.91
	Worst	1472.816	1779.22	2200.435	2845.624	1648.377	2797.678	2935.651	2062.389	1738.377	2591.357	2916.871	2473.996	2212.947
	Std	135.4067	219.8764	295.2874	242.7758	186.9812	263.5195	332.5847	215.659	133.4848	313.057	335.5618	352.4818	323.6579
	Median	1355.166	1445.056	2092.754	2403.414	1410.669	2310.509	2438.88	1781.838	1521.107	2334.287	2636.871	2022.487	1733.197
	Rank	1	3	7	11	2	10	12	6	4	9	13	8	5
C17-F11	Mean	1101.951	1140.586	1228.49	2875.633	1161.199	3483.1	1283.95	1128.6	1140.581	1166.078	1143.474	1158.09	2498.755
	Best	1100.106	1123.786	1148.587	2212.166	1146.001	1239.695	1142.653	1114.45	1125.089	1151.928	1134.302	1145.891	1127.276
	Worst	1103.709	1166.606	1396.414	3651.557	1172.502	5750.695	1486.99	1150.863	1155.683	1189.225	1150.141	1181.344	6389.56
	Std	1.471866	20.62707	113.236	604.7776	13.25433	2511.116	152.6594	15.59771	13.5372	16.09987	6.99637	15.96627	2594.499
	Median	1101.994	1135.977	1184.48	2819.403	1163.147	3471.005	1253.078	1124.544	1140.775	1161.58	1144.726	1152.563	1239.093
	Rank	1	4	9	12	7	13	10	2	3	8	5	6	11
C17-F12	Mean	1236.271	5559.916	2,511,388	2.22 × 10^8^	359,149.5	273,948.3	8,393,395	183,943.7	1,538,474	5,480,627	532,151.5	8674.094	656,172.8
	Best	1200.472	2570.303	1,341,217	72,092,500	73,415.04	90,941.23	1,034,605	53,497.94	352,069	1,466,612	87,116.48	2632.812	189,991.5
	Worst	1320.393	8481.589	4,328,205	3.96 × 10^8^	782,590.9	369,792.4	18,669,650	405,280	2,144,531	9,702,434	1,174,998	14,989.43	1,158,399
	Std	56.3488	2534.165	1,377,237	1.54 × 10^8^	302,391.3	128,159.5	7,419,726	153,869.8	829,555.8	4,362,453	490,016	5630.259	397,641.8
	Median	1212.11	5593.885	2,188,065	2.1 × 10^8^	290,296.1	317,529.7	6,934,662	138,498.5	1,828,648	5,376,730	433,245.8	8537.066	638,150.3
	Rank	1	2	10	13	6	5	12	4	9	11	7	3	8
C17-F13	Mean	1304.993	1344.367	8117.328	14,715,489	8085.628	7104.87	21,009.24	23,961.97	13,686.04	18,044.16	11,826.33	7084.083	58,962.19
	Best	1300.267	1326.243	4012.739	562,966.1	6942.551	3435.632	8238.108	1430.428	1754.981	17,033.29	9912.376	2482.891	9169.346
	Worst	1307.311	1388.356	12,303.06	38,809,343	8625.08	9713.084	33,878.59	32,971.52	29,492.54	20,513.96	13,294.02	18,031.96	195,190.8
	Std	3.216697	29.52882	3426.914	18,055,269	789.815	3079.479	11,270.67	15,062.17	12,680.32	1662.195	1404.497	7379.557	90,872.29
	Median	1306.198	1331.435	8076.757	9,744,822	8387.44	7635.382	20,960.14	30,722.97	11,748.31	17,314.69	12,049.47	3910.74	15,744.32
	Rank	1	2	6	13	5	4	10	11	8	9	7	3	12
C17-F14	Mean	1402.488	1444.236	2289.895	4231.887	1521.929	2518.19	2024.33	1458.924	2213.02	1621.184	7031.375	3146.623	13,967.98
	Best	1400.997	1436.972	1480.121	1776.823	1465.868	1498.802	1555.677	1451.275	1520.805	1540.045	4068.107	1449.32	3940.504
	Worst	1404.975	1461.52	4154.407	5069.283	1606.812	5475.471	2659.533	1467.14	4230.062	1654.052	9502.841	7325.205	27,939.94
	Std	1.722924	11.57122	1251.151	1636.771	63.50277	1971.796	461.422	8.566456	1344.803	54.35347	2875.255	2808.222	10,166.57
	Median	1401.99	1439.226	1762.525	5040.72	1507.517	1549.244	1941.054	1458.639	1550.606	1645.319	7277.277	1905.983	11,995.75
	Rank	1	2	8	11	4	9	6	3	7	5	12	10	13
C17-F15	Mean	1500.735	1543.281	6262.746	11,927.86	5086.004	8164.765	10,192.3	3355.232	8109.99	1742.133	22,752.48	9655.325	4826.672
	Best	1500.42	1516.603	2595.687	7176.916	3413.436	1622.124	2332.897	1548.837	1626.364	1606.337	9801.267	3004.24	1939.079
	Worst	1501.47	1576.696	10,895	18,873.62	5725.449	23,941.47	19,122.93	6441.311	13,816.11	1839.486	32,087.12	15,949.83	8587.927
	Std	0.492915	28.38924	3620.641	5484.158	1118.04	10,590.53	6882.544	2332.862	5279.614	114.4205	10,813.18	5410.479	3305.625
	Median	1500.525	1539.914	5780.148	10830.45	5602.566	3547.731	9656.686	2715.391	8498.744	1761.354	24,560.77	9833.616	4389.841
	Rank	1	2	7	12	6	9	11	4	8	3	13	10	5
C17-F16	Mean	1601.491	1649.923	1821.649	2117.474	1750.931	1902.667	1820.004	1954.402	1757.887	1699.423	2169.685	1967.285	1835.615
	Best	1600.891	1618.631	1746.382	1931.724	1626.595	1703.917	1662.416	1861.547	1676.507	1671.229	2002.795	1857.47	1744.823
	Worst	1602.221	1740.889	1927.75	2277.287	1870.188	2204.793	1928.273	2089.957	1886.671	1758.351	2292.979	2140.708	1869.332
	Std	0.559227	60.65097	88.96065	175.2947	99.64392	225.6027	127.3475	111.8741	96.67836	40.60161	121.7196	131.2036	60.58069
	Median	1601.426	1620.087	1806.232	2130.443	1753.471	1850.979	1844.663	1933.052	1734.186	1684.057	2191.484	1935.481	1864.152
	Rank	1	2	7	12	4	9	6	10	5	3	13	11	8
C17-F17	Mean	1723.586	1765.069	1823.242	1893.54	1766.873	1888.486	1865.574	1798.636	1761.392	1780.403	1845.77	1773.865	1777.796
	Best	1720.806	1743.094	1789.168	1820.852	1763.401	1786.601	1786.288	1748.661	1747.335	1769.398	1767.287	1766.627	1774.404
	Worst	1726.376	1777.321	1887.088	1950.365	1769.946	2028.162	1929.496	1821.921	1771.594	1791.214	2058.763	1781.135	1780.448
	Std	2.675517	15.11684	46.17573	62.33027	3.283575	109.6898	70.76013	33.79072	10.16705	10.80578	142.2006	6.203813	2.734527
	Median	1723.581	1769.93	1808.356	1901.471	1767.072	1869.592	1873.255	1811.981	1763.319	1780.5	1778.515	1773.849	1778.167
	Rank	1	3	9	13	4	12	11	8	2	7	10	5	6
C17-F18	Mean	1800.837	1859.672	13,938.97	59,571,750	3535.842	22,783.74	11,831.85	17,169.54	28,339.11	31,819.69	6760.801	23,558.67	13,746.07
	Best	1800.382	1831.441	7463.635	1,172,817	2282.239	7557.654	4843.622	3049.12	6350.854	25,849.48	2784.765	2988.52	3590.053
	Worst	1801.23	1885.454	30,792.79	2.31 × 10^8^	6112.985	38,664.33	18,444.13	28,482.17	43,862.39	39,830.07	11,735.05	43,985.86	19,884.62
	Std	0.425238	23.75548	11,291.02	1.14 × 10^8^	1769.324	17,002.7	5997.927	11,063.84	16,266.21	6430.793	3719.078	21,164.6	7117.107
	Median	1800.869	1860.897	8749.724	2,951,840	2874.071	22,456.49	12,019.82	18,573.44	31,571.61	30,799.6	6261.693	23,630.15	15,754.81
	Rank	1	2	7	13	3	9	5	8	11	12	4	10	6
C17-F19	Mean	1900.699	1926.294	17,532.55	887,210	2810.952	69,696.01	91,329.02	2061.048	9918.311	4947.729	37,009.45	26,876.64	6556.993
	Best	1900.02	1920.536	11,947.26	261,858.1	1991.015	1989.788	2432.302	1934.73	1948.214	2074.039	19,605.58	2703.715	2258.266
	Worst	1901.018	1936.758	22,949.02	1,457,677	4443.733	269,653.5	302,603.7	2186.265	14,970.35	13,386.75	56,169.17	83,121.28	10,563.68
	Std	0.469791	7.217362	4527.227	569,116	1113.981	133,312.5	141,491.7	138.5845	5844.748	5626.219	16,830.68	37,918.21	3426.682
	Median	1900.878	1923.94	17,616.97	914,652.3	2404.53	3570.371	30,140.06	2061.599	11,377.34	2165.063	36,131.53	10,840.78	6703.014
	Rank	1	2	8	13	4	11	12	3	7	5	10	9	6
C17-F20	Mean	2012.062	2054.989	2168.699	2315.007	2062.648	2354.534	2225.473	2091.528	2084.559	2097.948	2297.8	2202.964	2074.353
	Best	2000.995	2041.52	2097.348	2255.245	2054.132	2248.846	2216.119	2060.579	2060.809	2086.007	2211.927	2176.839	2058.758
	Worst	2022.277	2065.326	2289.042	2384.066	2075.653	2525.444	2244.977	2174.014	2122.146	2109.232	2418.189	2237.451	2082.762
	Std	10.64651	11.14546	85.72215	54.87906	9.167641	130.7472	13.17126	55.19227	26.7293	9.737469	99.95301	30.12514	11.06745
	Median	2012.488	2056.554	2144.203	2310.359	2060.403	2321.923	2220.398	2065.759	2077.64	2098.276	2280.541	2198.783	2077.947
	Rank	1	2	8	12	3	13	10	6	5	7	11	9	4
C17-F21	Mean	2227.269	2277.628	2303.89	2376.87	2277.037	2324.678	2318.231	2314.057	2340.214	2330.032	2385.654	2350.741	2328.384
	Best	2200	2223.137	2225.887	2290.952	2222.014	2228.428	2261.055	2222.011	2328.288	2226.03	2365.778	2342.005	2250.78
	Worst	2309.074	2332.025	2390.796	2417.574	2333.006	2422.937	2380.97	2356.142	2347.139	2371.958	2397.662	2358.925	2365.929
	Std	54.53713	61.08269	90.13716	57.92822	63.53908	103.91	63.56486	61.89353	8.23251	69.83162	13.80538	8.322173	52.39665
	Median	2200	2277.675	2299.439	2399.477	2276.563	2323.674	2315.448	2339.036	2342.715	2361.069	2389.588	2351.016	2348.414
	Rank	1	3	4	12	2	7	6	5	10	9	13	11	8
C17-F22	Mean	2281.681	2332.152	2330.768	3087.845	2329.939	2718.571	2342.642	2327.38	2334.197	2344.22	2323	2337.385	2342.437
	Best	2225.162	2327.867	2325.755	2817.831	2325.678	2264.186	2335.826	2326.342	2324.894	2337.414	2323	2323.692	2339.298
	Worst	2300.816	2337.065	2342.905	3336.034	2333.863	3182.255	2350.877	2328.435	2348.562	2356.955	2323	2372.308	2347.267
	Std	37.67967	3.848159	8.161645	229.8799	3.910382	445.6178	6.479651	0.855293	11.30366	8.937244	1.82 × 10^−10^	23.3312	3.403963
	Median	2300.372	2331.838	2327.207	3098.757	2330.107	2713.922	2341.933	2327.371	2331.666	2341.256	2323	2326.771	2341.592
	Rank	1	6	5	13	4	12	10	3	7	11	2	8	9
C17-F23	Mean	2611.357	2579.308	2667.971	2718.43	2637.237	2697.98	2694.535	2643.4	2646.047	2672.2	2786.48	2674.092	2686.975
	Best	2608.305	2323.003	2650.779	2706.877	2629.339	2655.067	2677.464	2633.778	2636.214	2660.434	2715.86	2666.127	2665.349
	Worst	2616.532	2672.945	2699.707	2741.376	2640.62	2748.004	2714.439	2650.725	2653.039	2682.164	2952.25	2687.118	2696.114
	Std	3.64721	170.9946	21.67124	15.74796	5.317777	41.0529	15.28266	7.839137	8.395193	9.651696	111.046	9.466652	14.63718
	Median	2610.296	2660.642	2660.699	2712.733	2639.494	2694.424	2693.118	2644.548	2647.467	2673.1	2738.904	2671.562	2693.219
	Rank	2	1	6	12	3	11	10	4	5	7	13	8	9
C17-F24	Mean	2500	2654.765	2810.582	2882.259	2762.108	2806.98	2824.644	2770.844	2779.167	2792.961	2608.498	2803.53	2757.421
	Best	2500	2525.074	2790.184	2864.037	2748.444	2669.506	2794.738	2767.398	2761.584	2788.468	2525	2784.989	2548.505
	Worst	2500	2779.422	2842.774	2898.174	2767.624	2899.038	2857.29	2779.739	2806.256	2797.165	2858.992	2818.582	2845.439
	Std	0.000208	142.3299	23.04341	15.24674	9.138715	98.25782	25.82208	5.959332	19.47309	3.56214	166.996	14.18211	139.8829
	Median	2500	2657.281	2804.685	2883.413	2766.182	2829.688	2823.274	2768.118	2774.413	2793.106	2525	2805.275	2817.869
	Rank	1	3	11	13	5	10	12	6	7	8	2	9	4
C17-F25	Mean	2897.743	2939.205	3002.932	3379.608	2951.006	3084.264	2923.738	2950.689	2967.678	2962.932	2961.387	2951.867	2983.245
	Best	2897.743	2926.72	2978.251	3289.304	2926.744	2978.771	2784.028	2926.876	2942.501	2943.1	2926.92	2927.701	2970.614
	Worst	2897.743	2974.732	3054.607	3454.308	2974.394	3341.814	2987.511	2975.572	2976.797	2982.504	2972.89	2976.076	2993.868
	Std	3.36 × 10^−8^	23.70208	35.87615	70.12321	26.94928	173.2479	96.18849	27.35035	16.79278	20.94847	22.97801	27.38743	9.901458
	Median	2897.743	2927.683	2989.435	3387.41	2951.444	3008.235	2961.706	2950.154	2975.707	2963.062	2972.869	2951.845	2984.249
	Rank	1	3	11	13	5	12	2	4	9	8	7	6	10
C17-F26	Mean	2825.003	2972.504	3385.253	4092.298	2831.005	3908.684	4085.422	3257.318	3200.927	3262.013	3220.902	2933.409	2925.977
	Best	2800.002	2828.894	3084.199	3766.19	2830.358	3507.432	3146.636	2929.126	2929.209	2942.092	2828	2828	2719.842
	Worst	2900	3164.947	4136.214	4330.297	2831.923	4302.23	4744.193	4241.866	3841.664	3988.718	4399.609	3047.636	3156.644
	Std	49.99818	169.2791	505.5009	237.3648	0.672201	432.8631	683.3607	656.3655	429.2242	487.656	785.8045	89.81023	221.2334
	Median	2800.005	2948.087	3160.299	4136.353	2830.87	3912.537	4225.429	2929.14	3016.417	3058.621	2828	2929	2913.711
	Rank	1	5	10	13	2	11	12	8	6	9	7	4	3
C17-F27	Mean	3089.302	3187.335	3131.406	3230.803	3126.61	3213.757	3170.819	3123.865	3127.21	3148.188	3285.324	3170.977	3196.967
	Best	3088.978	3138.891	3125.805	3163.998	3124.072	3185.351	3123.898	3120.635	3123.737	3126.785	3274.05	3128.643	3152.806
	Worst	3089.706	3220.632	3135.892	3360.837	3127.949	3243.35	3278.907	3126.25	3134.393	3209.196	3292.888	3222.369	3260.97
	Std	0.366278	34.63162	5.15301	88.33327	1.727664	28.24835	72.58966	2.388077	4.967803	40.68205	8.30382	39.41506	45.7305
	Median	3089.262	3194.909	3131.964	3199.189	3127.209	3213.163	3140.235	3124.288	3125.354	3128.386	3287.178	3166.448	3187.046
	Rank	1	9	5	12	3	11	7	2	4	6	13	8	10
C17-F28	Mean	3100	3209.471	3291.862	3805.864	3297.406	3485.689	3402.419	3231.942	3444.978	3375.163	3508.536	3354.094	3289.755
	Best	3100	3131.001	3131	3641.298	3131.318	3249.761	3206.373	3131.128	3417.601	3254.631	3448	3214.648	3179.683
	Worst	3100	3249.505	3445.94	4085.063	3480.946	3689.471	3510.723	3417.587	3468.617	3446.202	3545.106	3446.173	3579.424
	Std	7.84 × 10^−5^	55.48586	132.2739	199.9835	192.2555	180.4896	134.1804	135.448	20.93876	91.4386	42.94438	104.9692	193.8496
	Median	3100	3228.689	3295.254	3748.547	3288.68	3501.763	3446.29	3189.526	3446.846	3399.909	3520.519	3377.778	3199.956
	Rank	1	2	5	13	6	11	9	3	10	8	12	7	4
C17-F29	Mean	3145.635	3198.911	3328.492	3371.819	3223.14	3336.276	3401.823	3295.223	3208.039	3250.921	3362.277	3308.944	3277.638
	Best	3136.956	3178.603	3221.613	3307.382	3211.292	3249.631	3291.368	3232.601	3191.878	3200.04	3269.198	3202.464	3224.531
	Worst	3153.72	3210.792	3490.816	3407.078	3243.25	3490.938	3542.404	3343.345	3228.417	3275.391	3560.442	3398.811	3331.133
	Std	8.915623	14.73388	126.0596	44.12179	14.06066	109.1835	104.2167	52.40041	15.19086	35.35944	133.6847	89.23126	44.81565
	Median	3145.932	3203.125	3300.77	3386.407	3219.01	3302.268	3386.76	3302.473	3205.931	3264.127	3309.733	3317.25	3277.445
	Rank	1	2	9	12	4	10	13	7	3	5	11	8	6
C17-F30	Mean	3399.757	10,068.96	287,700.2	11,930,005	54,083.49	811,693.2	1,282,284	394,299.5	786,216.7	65,324.3	934,558	418,537.2	1,651,370
	Best	3395.483	4233.284	30,695.17	1,938,209	32,506.21	20,261.8	230,760.6	16,058.25	6350.293	31,434.05	576,418.6	6669.239	568,362.1
	Worst	3406.359	23,879.15	684,228	31,239,242	77,137.33	1,695,201	304,9926	1,493,937	2,907,196	109,796.9	1,285,469	830,093.5	3,762,394
	Std	4.745776	9332.561	317,094.7	13,174,546	24,872.51	910,839.9	1,321,431	733,263.4	1,416,501	38,272.74	289,499.1	474,595.8	1,505,564
	Median	3398.593	6081.714	217,938.8	7,271,284	53,345.21	765,654.9	924,224.8	33,601.32	115,660.1	60,033.11	938,172.3	418,693	1,137,363
	Rank	1	2	5	13	3	9	11	6	8	4	10	7	12
Sum rank		30	101	221	363	113	301	278	148	187	222	243	208	224
Mean rank		1.034483	3.482759	7.62069	12.51724	3.896552	10.37931	9.586207	5.103448	6.448276	7.655172	8.37931	7.172414	7.724138
Total rank		1	2	7	13	3	12	11	4	5	8	10	6	9

**Table 6:** Wilcoxon rank sum test results.

Compared Algorithm	Objective Function Type			
	Unimodal	High-Dimensional	Fixed-Dimensional	CEC 2017
SABO vs. WSO	1.08 × 10^−24^	1.97 × 10^−21^	0.000113	1.72 × 10^−19^
SABO vs. AVOA	0.057751	1.71 × 10^−5^	1.27 × 10^−18^	1.97 × 10^−21^
SABO vs. RSA	1.11 × 10^−5^	5.15 × 10^−11^	1.44 × 10^−34^	1.97 × 10^−21^
SABO vs. MPA	1.01 × 10^−24^	6.98 × 10^−15^	1.02 × 10^−8^	1.22 × 10^−18^
SABO vs. TSA	1.01 × 10^−24^	1.28 × 10^−19^	1.44 × 10^−34^	2.41 × 10^−21^
SABO vs. WOA	1.01 × 10^−24^	5.16 × 10^−14^	1.44 × 10^−34^	5.93 × 10^−21^
SABO vs. MVO	1.01 × 10^−24^	1.97 × 10^−21^	1.44 × 10^−34^	1.16 × 10^−20^
SABO vs. GWO	1.01 × 10^−24^	7.58 × 10^−16^	1.44 × 10^−34^	1.97 × 10^−21^
SABO vs. TLBO	1.01 × 10^−24^	1.04 × 10^−14^	1.44 × 10^−34^	7.05 × 10^−21^
SABO vs. GSA	1.01 × 10^−24^	1.97 × 10^−21^	1.46 × 10^−13^	2.13 × 10^−21^
SABO vs. PSO	1.01 × 10^−24^	1.97 × 10^−21^	1.2 × 10^−16^	1.97 × 10^−21^
SABO vs. GA	1.01 × 10^−24^	1.97 × 10^−21^	1.44 × 10^−34^	2.09 × 10^−20^

**Table 7:** Performance of optimization algorithms for the pressure vessel design problem.

Algorithm	Optimum Variables				Optimum Cost
	*T_s_*	*T_h_*	*R*	*L*	
SABO	0.778027	0.384579	40.31228	200	5882.901
WSO	0.778027	0.384579	40.31228	200	5882.901
AVOA	0.778027	0.384579	40.31228	200	5882.901
RSA	0.802584	0.844696	40.70183	200	7482.575
MPA	0.778027	0.384579	40.31228	200	5882.901
TSA	0.779501	0.39248	40.31357	200	5916.63
WOA	0.829716	0.514131	41.05723	189.8821	6541.663
MVO	0.857853	0.42615	44.43659	149.6475	6044.237
GWO	0.780028	0.386828	40.41563	198.6128	5891.034
TLBO	1.203325	1.486222	58.51898	69.96653	14,118.06
GSA	1.227629	0.698407	51.74761	114.4765	9945.211
PSO	1.333518	1.047126	67.93357	17.51237	12,075.36
GA	1.475349	0.666431	53.45493	162.9081	14,813.53

**Table 8:** Statistical results of optimization algorithms for the pressure vessel design problem.

Algorithm	Mean	Best	Worst	Std	Median	Rank
SABO	5882.901	5882.901	5882.901	1.87 × 10^−12^	5882.901	1
WSO	5915.105	5882.901	6498.223	137.3202	5882.901	3
AVOA	6481.485	5883.345	7313.806	539.7058	6320.198	6
RSA	13,096.94	7482.575	31,153.65	5269.559	11,801.01	9
MPA	5882.901	5882.901	5882.901	7.88 × 10^−6^	5882.901	2
TSA	6338.723	5916.63	7390.013	508.4069	6070.166	5
WOA	8510.284	6541.663	11,984.76	1564.63	7876.801	8
MVO	6728.999	6044.237	7328.396	420.5985	6712.525	7
GWO	6025.596	5891.034	7223.113	382.0974	5903.094	4
TLBO	29,784.06	14,118.06	54,039.67	10,010.76	29,098.17	11
GSA	22,548.81	9945.211	40,013.2	8166.03	21,797.25	10
PSO	32,641.1	12,075.36	74,979.4	18,333.66	31,455.76	12
GA	32,672.62	14,813.53	57,925.8	12,038.98	30,822.44	13

**Table 9:** Performance of optimization algorithms for the speed reducer design problem.

Algorithm	Optimum Variables							Optimum Cost
	*b*	*M*	*p*	*l*_1_	*l*_2_	*d*_1_	*d*_2_	
SABO	3.5	0.7	17	7.3	7.8	3.350215	5.286683	2996.348
WSO	3.5	0.7	17	7.300011	7.800021	3.350215	5.286686	2996.349
AVOA	3.5	0.7	17	7.3	7.8	3.350215	5.286683	2996.348
RSA	3.6	0.7	17	8.3	8.3	3.367585	5.5	3201.663
MPA	3.5	0.7	17	7.3	7.8	3.350215	5.286683	2996.348
TSA	3.502148	0.7	17	7.3	8.3	3.35245	5.289842	3010.76
WOA	3.5	0.7	17	7.3	7.8	3.367938	5.291747	3004.112
MVO	3.512969	0.7	17	7.531103	7.8	3.358073	5.28743	3005.97
GWO	3.500135	0.7	17	7.465414	7.842208	3.351387	5.288783	3000.422
TLBO	3.580555	0.702711	24.74533	8.098778	8.176551	3.674643	5.412883	4887.56
GSA	3.542686	0.702648	17.21175	7.499948	7.843232	3.588004	5.320297	3152.102
PSO	3.540769	0.70174	27.65403	7.555885	8.17207	3.390954	5.389825	5497.948
GA	3.554445	0.706553	20.58122	7.559935	8.141695	3.627213	5.383383	3897.082

**Table 10:** Statistical results of optimization algorithms for the speed reducer design problem.

Algorithm	Mean	Best	Worst	Std	Median	Rank
SABO	2996.348	2996.348	2996.348	9.33 × 10^−13^	2996.348	1
WSO	2996.428	2996.349	2997.378	0.229195	2996.36	3
AVOA	3001.508	2996.348	3012.836	4.579725	3001.278	4
RSA	3275.755	3201.663	3363.128	58.7856	3268.023	9
MPA	2996.348	2996.348	2996.348	1.03 × 10^−5^	2996.348	2
TSA	3031.051	3010.76	3055.05	12.61348	3031.917	7
WOA	3119.79	3004.112	3241.885	71.0998	3139.051	8
MVO	3027.597	3005.97	3055.36	14.62276	3028.79	6
GWO	3005.626	3000.422	3015.259	4.261653	3005.579	5
TLBO	9.03 × 10^13^	4887.56	3.39 × 10^14^	9.66 × 10^13^	5.47 × 10^13^	12
GSA	3622.122	3152.102	4409.364	332.2137	3665.23	10
PSO	1.67 × 10^14^	5497.948	5.21 × 10^14^	1.61 × 10^14^	1.37 × 10^14^	13
GA	4.37 × 10^13^	3897.082	1.77 × 10^14^	4.76 × 10^13^	2.62 × 10^13^	11

**Table 11:** Performance of optimization algorithms for the welded beam design problem.

Algorithm	Optimum Variables				Optimum Cost
	*h*	*l*	*t*	*b*	
SABO	0.20573	3.470489	9.036624	0.20573	1.724852
WSO	0.20573	3.470489	9.036624	0.20573	1.724852
AVOA	0.20573	3.470489	9.036624	0.20573	1.724852
RSA	0.168536	4.097767	10	0.204452	1.908712
MPA	0.20573	3.470489	9.036624	0.20573	1.724852
TSA	0.20487	3.485327	9.06275	0.206144	1.733193
WOA	0.205398	3.46205	9.077283	0.21425	1.795184
MVO	0.204071	3.502486	9.058767	0.205639	1.729722
GWO	0.20563	3.472437	9.041285	0.205727	1.725749
TLBO	0.366925	3.230843	8.472588	0.399745	3.288168
GSA	0.269422	2.818837	7.907051	0.269422	1.94981
PSO	0.407489	5.001097	5.120335	0.644527	3.934221
GA	0.152949	6.850027	7.076448	0.44196	3.314212

**Table 12:** Statistical results of optimization algorithms for the welded beam design problem.

Algorithm	Mean	Best	Worst	Std	Median	Rank
SABO	1.724852	1.724852	1.724852	6.83 × 10^−16^	1.724852	1
WSO	1.724859	1.724852	1.724984	2.94 × 10^−5^	1.724852	3
AVOA	1.766698	1.724892	1.894369	0.047302	1.749276	7
RSA	2.206838	1.908712	2.432765	0.167509	2.206361	8
MPA	1.724852	1.724852	1.724852	9.33 × 10^−9^	1.724852	2
TSA	1.743398	1.733193	1.753578	0.005756	1.742708	5
WOA	2.507115	1.795184	4.863039	0.864664	2.221353	10
MVO	1.744239	1.729722	1.766106	0.010415	1.740333	6
GWO	1.727645	1.725749	1.731538	0.001804	1.726748	4
TLBO	8.86 × 10^12^	3.288168	1.06 × 10^14^	2.67 × 10^13^	5.130472	12
GSA	2.444295	1.94981	3.206643	0.318079	2.420274	9
PSO	3.23 × 10^13^	3.934221	1.47 × 10^14^	5.01 × 10^13^	2.52 × 10^12^	13
GA	5.15 × 10^12^	3.314212	9.81 × 10^13^	2.19 × 10^13^	5.32709	11

**Table 13:** Performance of optimization algorithms for the tension/compression spring design problem.

Algorithm	Optimum Variables			Optimum Cost
	*d*	*D*	*p*	
SABO	0.051689	0.356718	11.28897	0.012665
WSO	0.051689	0.356718	11.28894	0.012665
AVOA	0.051689	0.356718	11.28897	0.012665
RSA	0.05	0.31073	15	0.013206
MPA	0.051688	0.35669	11.29061	0.012665
TSA	0.052552	0.377537	10.18444	0.012704
WOA	0.050879	0.337552	12.50787	0.012677
MVO	0.060316	0.601884	4.373275	0.013955
GWO	0.050839	0.33652	12.59396	0.012694
TLBO	0.069085	0.936567	2	0.01788
GSA	0.054593	0.420665	8.807168	0.013549
PSO	0.055083	0.362868	14.11723	0.017745
GA	0.069092	0.936121	2	0.017875

**Table 14:** Statistical results of optimization algorithms for the tension/compression spring design problem.

Algorithm	Mean	Best	Worst	Std	Median	Rank
SABO	0.012665	0.012665	0.012665	1.32 × 10^−18^	0.012665	1
WSO	0.01268	0.012665	0.01278	2.79 × 10^−5^	0.012671	3
AVOA	0.013025	0.012691	0.014417	0.000435	0.012881	6
RSA	0.021483	0.013206	0.105648	0.023175	0.013311	11
MPA	0.012665	0.012665	0.012665	3.25 × 10^−8^	0.012665	2
TSA	0.012951	0.012704	0.013815	0.000279	0.012868	5
WOA	0.013608	0.012677	0.015812	0.000964	0.013087	7
MVO	0.017091	0.013955	0.01811	0.001373	0.017875	8
GWO	0.012746	0.012694	0.013145	9.55 × 10^−5^	0.012725	4
TLBO	0.018531	0.01788	0.01934	0.000379	0.01848	9
GSA	0.019326	0.013549	0.02727	0.003827	0.019966	10
PSO	2.98 × 10^13^	0.017745	3.97 × 10^14^	9.71 × 10^13^	0.017773	13
GA	1.08 × 10^12^	0.017875	2.09 × 10^13^	4.66 × 10^12^	0.023851	12

## Data Availability

Not applicable.
